# From local knowledge and science to policy: Lessons learned from Fiji's valuable grouper fisheries

**DOI:** 10.1111/jfb.16041

**Published:** 2025-01-08

**Authors:** Yvonne Sadovy de Mitcheson, Aisake Batibasaga, Chloe E. R. Hatten, Sangeeta Mangubhai

**Affiliations:** ^1^ Division of Ecology and Biodiversity, School of Biological Sciences The University of Hong Kong Pok Fu Lam Hong Kong; ^2^ Fiji Fisheries Department, Research Division Lami Fiji; ^3^ Fiji Country Program Wildlife Conservation Society Suva Fiji; ^4^ Present address: IUCN Groupers & Wrasses Specialist Group and Science and Conservation of Fish Aggregations; ^5^ Present address: ISA Coastal Fisheries Management and Conservation Consultancy Nasinu Fiji; ^6^ Present address: Talanoa Consulting Suva Fiji

**Keywords:** aggregations, coastal, conservation, management, Pacific, reef

## Abstract

Pacific Island communities are heavily dependent on fisheries for subsistence and livelihoods. Yet, despite their importance, coastal fisheries are poorly managed and commercial pressures increasingly threaten them. Groupers (Epinephelidae) are exceptionally vulnerable to overexploitation due to aspects of their biology while their economic value makes them a prime target for commerce. Fiji has a significant grouper fishery and is a useful case study to assess a data‐poor, economically valuable sector to evaluate management measures, options, and needs. Data from multiple sources over three decades were integrated with original research involving fisher interviews, market surveys, stock assessments, and underwater census to assess the status of the country's grouper fishery. Catch rates are declining and trade now includes a high percentage of immature groupers, with aggregating species (mainly *Epinephelus polyphekadion*, *Epinephelus fuscoguttatus*, *Plectropomus areolatus*, *Plectropomus leopardus*) particularly at risk. Estimated annual grouper landings are increasing and now exceed 1000 mt. There is an urgent need to update Fiji's grouper size limits which are grossly inadequate. To build public support and increase awareness, government and nongovernmental organizations should invest in the national 4FJ Fish Smart campaign. Key management recommendations for groupers are (1) improved spatial and temporal protection of spawning aggregations and (2) increased minimum‐size restrictions for capture and sale. Findings apply broadly to valuable and vulnerable coastal fin‐fisheries in reef ecosystems across many Pacific Island countries and highlight the importance of using multiple data sources and approaches to understand and manage important data‐poor fisheries.

## 1 INTRODUCTION

Pacific Island coastal fisheries include a diversity of fishes and invertebrates used for subsistence and commercial purposes (e.g., Dalzell et al., 1996). Despite the cultural and socioeconomic importance of coastal fisheries for most Pacific Island communities (ibid.), surprisingly little government attention is directed toward their management, especially when compared to offshore fisheries. This is despite the existence of regional policy frameworks to coordinate and guide sustainable fisheries development (e.g., A New Song for Coastal Fisheries—Pathways to Change: The Noumea Strategy, Pacific Islands Regional Coastal Fisheries Management Policy), international commitments (e.g., Sustainable Development Goals), and notwithstanding alarming declines in many coastal resources in the region.

Fiji is one of the biggest island nation economies in the Pacific with significant coastal commercial and subsistence fishery sectors, a long history of marine resource exports, and a strong tourism market for both seafood and eco‐activities (Gillett & Fong, 2023). Its fisheries are relatively well studied and its policies specifically recognize the need to consider the importance of fishing for employment and for improving the living standards of the rural population (Gillett, 2011). However, although coastal fisheries are important for trade and, particularly, for subsistence use, they are inadequately monitored or managed. To support its national development, Fiji joined the World Trade Organization in 1996, adopting an export‐oriented trade policy. However, this policy does not explicitly consider the long‐term impacts of export trade on its limited natural marine resources or for its rural communities. As such, the economic benefits of most coastal resource exports for the country are unknown and the implications of exports for resource status and local communities are not understood.

Demand for seafood in Fiji is high and, as elsewhere in the region, is growing due to an increasing population, expanding demand from tourism, and pressures to export (Bell et al., 2009; Gillett & Fong, 2023). Coastal fisheries remain a significant source of fresh high‐quality protein for the country and are critically important sources of food and income in times of crisis or disasters, from cyclones and political unrest to the COVID‐19 pandemic, when access to imported protein can be impeded (e.g., Ferguson et al., 2022). The percentage contribution of fresh fish to total fish consumption in Fiji was estimated at 59% across the country and 92% for coastal communities (Bell et al., 2009). Exports of coastal species for the ornamental fish and invertebrate trade, as well as beche‐de‐mer, among other taxa, are variously documented and are, or have been, significant (e.g., Gillett, 2011; Teh et al., 2009). Surprisingly little, however, is known about ongoing food fish exports from coastal fisheries. In 2018, seafood contributed at least 1.8% to the national gross domestic product (GDP), the third‐largest primary sector in Fiji (Ministry of Fisheries 2017–2018).

Fiji's coastal fisheries are now facing increasing threats with potentially serious consequences for Fijians under the *status quo* (Mangubhai et al., 2019). Although the export‐oriented offshore fisheries are relatively well managed and monitored, most coastal fisheries are severely underrepresented in national fisheries and related data for volumes and values. Between 1992 and 2012, coastal and offshore fishery sectors each produced comparable landings, between 11,000 and 27,000 mt annually, according to official figures. However, since the 1990s, the condition of coastal fisheries has steadily worsened (Fong, 1994; Gillett, 2009; Richards et al., 1994; Starkhouse, 2009), with an increasing proportion of fish and diversity of species being taken, overfished, and often sold before they reach adulthood (Lee et al., 2020; Prince et al., 2018, 2019; Sadovy de Mitcheson et al., 2018). The core fisheries legislation, the Fisheries Act 1941 (Cap 158), needs modernization (Minter, 2008) but the fisheries are data‐poor making management challenging to implement. At present rates of exploitation, Fiji may not be seafood secure by 2030 (Bell et al., 2009).

Groupers (family Epinephelidae) are among the most highly prized and economically important finfishes in the coastal fisheries of the country (Fox et al., 2012; Lee et al., 2020; Sadovy de Mitcheson et al., 2018). The biology of many groupers (i.e., longevity, slow growth, reproductive mode) can make them particularly susceptible to unmanaged fishing (e.g., Sadovy de Mitcheson, Linardich, et al., 2020). A specific example is the reproductive mode of aggregation spawning: reproductive gatherings typically briefly form each year and are the only times when adults come together at certain reef sites to spawn. Several medium‐to‐large groupers have historically been a seasonal target of traditional fishing across the Pacific because hundreds to thousands of adults migrate across the reef to gather predictably at specific locations annually yielding high catch rates during these times. Before commercial exploitation such species evidently withstood community fishing pressure for subsistence needs. However, commercialization and exploitation without controls can decimate spawning aggregations along with the fish populations (and fisheries outside of the spawning seasons and aggregations) that these support (Fox et al., 2012; Sadovy & Batibasaga, 2006; Sadovy de Mitcheson & Colin, 2012). Similar challenges are being experienced by many Pacific Island nations today.

The importance and vulnerability of groupers, especially aggregating species, should make them a high priority for policy and regulation across small‐scale fisheries of the tropics and subtropics (Sadovy de Mitcheson, Linardich, et al., 2020). However, although variously studied or documented, information on the taxon is often not readily available, being scattered among published and unpublished sources; sometimes, only fishers understand the resource they exploit. For multiple reasons, the grouper fisheries of Fiji are a prime candidate for a case study that assembles and integrates data from multiple decades and diverse sources to inform management. Here, we pay special attention to those grouper species that aggregate to spawn and consider their long‐term sustainability considering their status and current management profile. This work highlights the value of drawing on multiple and diverse information sources in a data‐poor and little monitored fishery with lessons applicable to many other island nations, particularly in the Pacific.

The objectives of this case study were to review both published and unpublished information and conduct novel research with fishers and in the field with a view to understanding the history, importance, and status of grouper fisheries in Fiji. Specifically, our aims were to (1) quantify the role of groupers in Fiji in terms of volumes, species, sizes, and seasonality of fish in trade; (2) determine perceived fisher trends in key aggregating grouper fisheries, including aggregation timing and location, and identify any associated concerns; and (3) conduct underwater surveys to validate interview outcomes and provide a baseline for future study at one aggregation site, Naiqoro Passage. Based on the findings measures for safeguarding the long‐term sustainable use of groupers were identified for Fiji and considered in the broader context of Pacific Island nations.

## 2 MATERIALS AND METHODS

Considering the importance of groupers to tropical and subtropical coastal fisheries and seafood trade (domestic and international), as well as the high vulnerability of the taxon to fishing, this study assembles and evaluates available information on groupers within Fiji from a wide range of data sources, and reports on novel research conducted to address data gaps. Particular attention is paid to major aggregating species of interest due to their high value and seasonal volumes and to the susceptibility of their spawning aggregations to fishing: *Epinephelus polyphekadion, Epinephelus fuscoguttatus, Plectropomus areolatus*, and *Plectropomus leopardus*.

### 2.1 Volumes, species, sizes, and seasonality of groupers in trade

For insights into the absolute and relative importance of groupers in Fiji's coastal fisheries, reports, publications, catch and market surveys, interviews, and government data were reviewed. In addition to estimates of commercial grouper trade by year, information was compiled on total coastal catches and combined with estimates of the proportion of catches comprising groupers as an alternative means of estimating annual grouper landings when these were not available. Grouper exports were estimated based on available data. Information is presented by species, when possible, and any indications of seasonality in catches or trade noted.

To estimate the volumes of grouper in annual catches and trade over three decades, we reviewed four sources of available information: (1) independent estimates of annual grouper catches; (2) volume of groupers calculated as a percentage of catches comprising groupers (i.e., applying the percentage of groupers in total catches that we calculated to indicate their relative importance); (3) government data from market surveys (1980–2008); and (4) value chain analysis (VCA) results involving 138 interviews of fishers, middlemen, hotels and restaurants, and exporters.

To understand sizes of grouper in trade and the implications of these for spawning potential, length data (fork length [FL] to the nearest mm) were collected and a stock assessment reviewed. Length data were documented from across municipal fish markets on the two main islands, Viti Levu (*n* = 369 fish) and Vanua Levu (*n* = 217), between 2016 and 2018. During each market visit, data were collected by trained surveyors from fish before or after sale when permission was granted. Whenever possible at least 50 individuals per species were dissected. Body sizes at maturity were determined macroscopically in the field from the appearance of the sex organs (gonads) and calculated by plotting the proportion of mature individuals in the population by length class (Prince et al., 2018, 2019, 2020). A stock assessment based on size data calculated the spawning potential ratio (SPR), that is, the proportion of the unfished reproductive potential left at any given level of fishing pressure. SPR is used to set target and limit reference points for fisheries, and an SPR of 20% is the international limit reference point above which fish stocks should be maintained to reduce the risk of stock decline (Goodyear, 1993). SPR 10% is the international reference point below which fish populations are expected to collapse (Prince et al., 2020).

### 2.2 Perceived trends in key aggregating grouper fisheries

**FIGURE 1:**
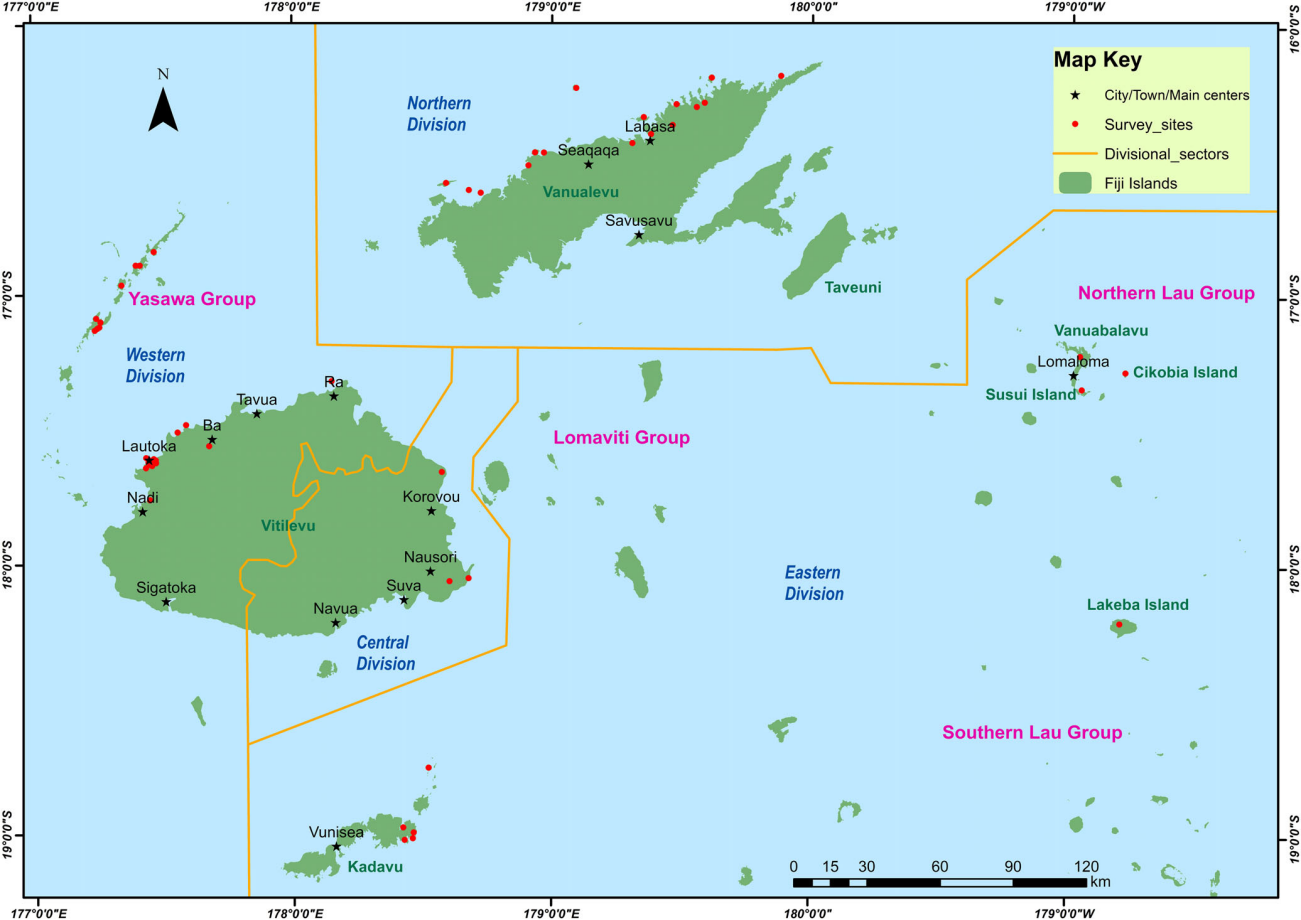
Sites (red dots) at which grouper fisher surveys were conducted between 2003 and 2017 and towns (text in black) indicated in the manuscript. Island groups are written in pink. The yellow lines demarcate four fishery sector divisions as designated by the Ministry of Fisheries (North, West, Central, East).

A total of 213 semi‐structured interviews were conducted between 2003 and 2017 across Fiji with *i‐Taukei* (Indigenous) and Indo‐Fijian fishers to understand their experiences and perceptions about groupers over time (decades of the 1970s, 1980s, 1990s, and into the 2000s), particularly with aggregating species. In October–November 2003, February 2004, and October 2005, 79 semi‐structured interviews were conducted on the islands of Vanua Levu, Lakeba, Viti Levu, the Yasawas, and Vanua Balavu. Between June and August 2008, 63 semi‐structured interviews were conducted on Viti Levu, Vanua Levu, and Kadavu (Kuridrani, 2008). Interviews were also conducted between September 2016 and April 2017 on Viti Levu and Vanua Levu. Communities were selected to reflect a wide range of locations and different levels of fishing pressure across Fiji focusing on areas where groupers are often taken in catches while also considering travel logistics and opportunity (Figure 1).

Interviews were conducted orally in English or Fijian (i‐Taukei) languages mostly with individual fishers in their homes and based on their language preference. Photos of fish, together with local fish names and maps associated with familiar local place names, aided in species identifications and spatial responses. Whenever possible, community leaders selected fishers with at least 10 years' experience, who fished regularly, targeted groupers among their catches, and depended on fishing for their livelihood. Fisher names were kept confidential. Some fishers (especially night divers) were reluctant to answer questions assuming the Ministry of Fisheries (conducting some of the interviews) would penalize them for poaching and for using destructive fishing practices. We acknowledge this potential bias as a limitation to the study.

Questions included fish species, catch per trip, perceived trends over time (increase, decrease, or no change), and fisher concerns, if any. Catch data were summarized on the perceived average catch per trip trends of main species taken with a focus on aggregating groupers identified: *E. polyphekadion, E. fuscoguttatus, P. areolatus*, and *P. leopardus*. To identify possible spawning timing and aggregation sites questions in 2003–2008 sought to identify locations, months, and lunar phases of highest catches, largest fish numbers seen underwater by spearfishers, and presence of multiple fish caught with eggs. These characteristics are among those that fishers might notice that typify the presence of a spawning aggregation (Sadovy de Mitcheson & Colin, 2012).

To account for the possible influence on responses of overall level of fishing pressure in different areas data were summarized spatially based on high, medium and low fishing pressure across the country. Fishing pressure levels by area were determined using both fishing license numbers and expert opinion as follows: high: Naviti, Viti Levu, and Kadavu; medium: Vanua Levu; low: Vanua Balavu and Lakeba. Interview data were not normally distributed, so Kruskal–Wallis and post hoc Dunn tests were performed. All data preparation, statistical analyses, and graphics were performed in R version 1.4.1106 (R Core Team, 2021).

### 2.3 Underwater surveys of Naiqoro aggregation site for interview validation and baseline case study

To help validate interview responses for timing and location of aggregations and species reported, it is advisable to conduct in‐water field studies (Colin et al., 2003; Sadovy de Mitcheson & Colin, 2012). In addition, detailed understanding of selected aggregations in terms of species, timing, and location is a valuable baseline against which to assess any changes over time including outcomes of management action.

Twenty‐four of the 63 interviews completed by Kuridrani (2008) across Fiji were conducted in five villages on Kadavu Island: Matasawalevu, Dravuni Island, Vacalea, Solotavui, and Nukuvou. Interviewees identified five historically exploited aggregations (Vanuakula, Namara, Vesi, Korolevu, and Naiqoro Passages) with only two still considered viable at the time of the interviews: Naiqoro Passage (near Matasawalevu) and Vanuakula Passage (near Dravuni Island). Naiqoro Passage was selected by the Ministry of Fisheries for baseline underwater surveys because of local community efforts in its protection and tourism interest. Visits were made to Matasawalevu to explain the purpose of the underwater surveys and to seek permission to conduct the research.

Naiqoro Passage is an outer reef channel in northeastern Kadavu, located within the *i‐Qoliqoli* (customary marine tenure over inshore areas) of Nakasaleka District. In 2002–2003, due to a long‐standing problem of poaching in the channel by outsiders, the passage was protected by Matasawalevu Village community members. Because the passage is also a popular tourist dive site a collaboration in protection had been arranged by local dive resorts that supplied fuel to the village to patrol the passage, assisted by the Fisheries Department, local police, and the Kadavu Yaubula (Natural Resource) Management Support Team.

Field surveys were conducted annually from 2009 to 2012, inclusive, during July and August, identified by fishers to be the main aggregation season for several groupers at Naiqoro Passage. Timing of surveys (i.e., months) and locations of aggregations were based on local knowledge and recommendations from the Ministry of Fisheries. Dives on scuba ran along the Passage walls and outer reef apron, extending both north and south along the seaward edges of the Passage entrance, to locate groupers and map the extent of their occurrence. Fisheries staff and other interested field‐workers (local NGOs, invited local and overseas researchers) assisted in the surveys and received training.

#### Sampling protocol

To delineate the full extent of the aggregation area to standardize subsequent fish counts a Global Positioning System Unit was towed on the surface in a waterproof housing (Colin et al., 2005) and preliminary dives conducted in 2008. Three divers surveyed the entire site for groupers with two dives each per day along deep and shallow transects. Divers swam slowly in parallel, separated by 15–20 m, 5–7 m above the substrate. Unless visibility was (rarely) poor they counted fish up to 10 m on either side of their predetermined swimming routes; they used a signaling system to avoid double‐counting of fish occurring midway between adjacent divers (Sadovy de Mitcheson, Colin, et al., 2020). The shallow survey transect covered a depth of between 10 and 20 m and the deeper transect between 20 and 30 m. Few fish were seen during exploratory surveys to deeper waters and a maximum swimming depth of 25 m safely allowed sufficient time for two dives per day.

#### Field surveys

A total of 55 dives that fully surveyed the site were conducted in July–August 2009, 2010, 2011, and 2012, in addition to preliminary dives in 2008 and rapid surveys in late August 2012. The survey protocol established in 2008 was applied in all years. Surveys were conducted in the morning and afternoon during different phases of the tide, whenever weather and field conditions permitted, with divers generally swimming with the current. Divers noted behaviors often indicative of imminent spawning, including frequent chasing, color changes, courtship, and the presence of gravid females. Because fisher interviews had not consistently identified a specific moon phase associated with aggregation, surveys were timed to cover all moon phases.

## 3 ETHICS STATEMENT

Ethical approval for fisher interviews was not required by either national laws (in Fiji) or by the University of Hong Kong for such work during the 2003–2005 studies. In each community in Fiji, local protocol was strictly followed for conducting interviews. Staff from the government Fisheries Department conducted a traditional protocol and obtained permission from community chiefs prior to entering each community. Introductions to fishers were made based on community recommendations. The purpose of the study was clearly explained to the community chief and each fisher and their responses were coded so that their identity could be determined at no point. In Fiji oral communication, rather than written communication, is the norm and this was done in either Fijian or English based on the preference of the interviewee. Animal welfare ethical approval was not necessary as we did not use experimental animals.

## 4 RESULTS

### 4.1 Volumes, species, sizes, and seasonality of groupers in trade

Data assembled provide ample evidence of the importance of groupers for Fiji's inshore catches in terms of total calculated and relative proportions of catch volumes, trends over time, and trade. Major species are identified with a seasonal component indicated for both catches and trade for some. Overall, despite the poor documentation of coastal fisheries in the country, in particular of subsistence landings, the outcomes across data sources are consistent and provide a useful profile of the taxon.

Groupers make up a small but important percentage of total coastal (subsistence and commercial) finfish landings and are particularly important in commercial landings. From 2002 to 2016, groupers comprised between 5% and 10% (midpoint of 7.5%) of total coastal landings (IAS‐USP, 2009; Ministry of Fisheries 2016–2017, 2017–2018). Of recorded commercial finfish landings sold across local markets in Fiji groupers comprised 12% (± 0.02%) based on government data from 1980 to 2008 (Ministry of Fisheries Annual Reports 2014–2021) (Supplementary Information S1). About 25–30 grouper species are taken, with 10 being most common. *Cephalopholis* and smaller *Epinephelus* spp. and individuals are used for subsistence and some domestic trade. Larger species and individuals of *Epinephelus* and *Plectropomus* are traded domestically, including for restaurants and hotels, as well as for export.

Estimates of total annual landings of grouper clearly suggest substantial increases over approximately three decades, even as catch per trip declined, and highlight important discrepancies among data sources. Estimates of annual grouper commercial catches (reported as “rock cod” = *Epinephelus* species) for 1986–1992 were 302–601 t (Richards et al., 1994). Applying the 7.5% grouper midpoint (see previous paragraph) to estimates of total annual coastal catches from 2007 to 2015 (i.e., between 17,777 and 27,000 mt; Dalzell et al., 1996; Starkhouse, 2009; Gillett, 2016) produced annual grouper landings estimates of 1333–2025 mt. According to government annual reports from 1980 to 2008 average recorded grouper sales in municipal and nonmunicipal markets were 507 mt (±62 mt). These data, however, substantially underestimate totals because many retail outlets were not sampled including smaller markets or direct sales to restaurants and hotels.

Finally, the grouper VCA estimated grouper catches at between 1335 and 1580 mt for the period 2016–2017. This assumes that a grouper bundle (a local unit of sale weight and that used in the VCA) weighs about 2–3 kg (Sadovy de Mitcheson et al., 2018; A. Batibasaga, personal communication 2024). Of particular interest for their high value (alphabetically) are *Epinephelus coeruleopunctatus, E. coioides, E. cyanopodus, E. fuscoguttatus, E. maculatus, E. malabaricus, E. polyphekadion, Plectropomus areolatus, P. laevis*, and *P. leopardus* (Sadovy de Mitcheson et al., 2018). According to recent government data of the 10 top inshore species by price in municipal markets 9 were groupers at USD5.8–7.5/kg (Ministry of Fisheries 2020–2021).

International trade in groupers was estimated using government data, trader records and interviews, and professional opinion. Estimated volumes exported ranged from 3 to 70 mt annually within the last few decades, with most exports occurring during or following the spawning season for major exported species. These figures come from several sources. (1) The annual export of live reef fishes (mainly groupers) from the mid‐1990s and early 2000s to Hong Kong and mainland China peaked at 21 mt annually in 2003 with about 75% of fish caught during the spawning season of June–October (Ovasisi, 2006; Yeeting et al., 2001). (2) Exports of groupers from Fiji to the United States between 1996 and 2003 (as determined by US import data) were annually from 3 to 17 mt, mostly from July–December (NMFS, 2016). (3) In 2013 and 2014, a single trader exported 21–22 mt of groupers annually (Gold Hold company data 2015). (4) Government export data between 2013 and 2021 ranged from 4 to 27 mt annually. These figures are considered minimal as available export data are incomplete and represent only about 10‐20% of grouper exports (Ministry of Fisheries Annual Reports 2014, 2016‐2017, 2017‐2018, 2018‐2019, 2020‐2021). (5) A VCA analysis provided a more complete indication of recent exports at 70 mt annually based on interviews of major traders (Sadovy de Mitcheson et al., 2018). Main export destinations are New Zealand, China, including Hong Kong, South Korea, and the United States.

**FIGURE 2:**
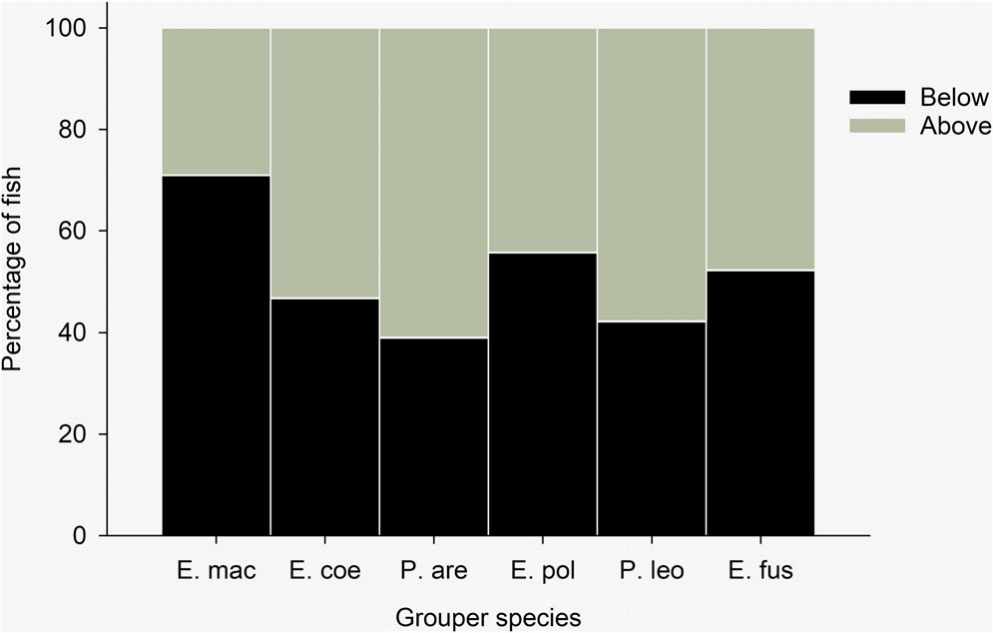
Percentage of fish above (gray) and below (black) relative to the size of maturity at municipal fish markets in Fiji. E. coe, *Epinephelus coeruleopunctatus*; E. fus, *Epinephelus fuscoguttatus*; E. pol, *Epinephelus polyphekadion*; E. mac, *Epinephelus maculatus*; P. are, *Plectropomus areolatus*; P. leo, *Plectropomus leopardus*. Data are pooled for the years 2016–2018. Source: Biospherics, Ministry of Fisheries, Wildlife Conservation Society, World Wide Fund for Nature.

**FIGURE 3:**
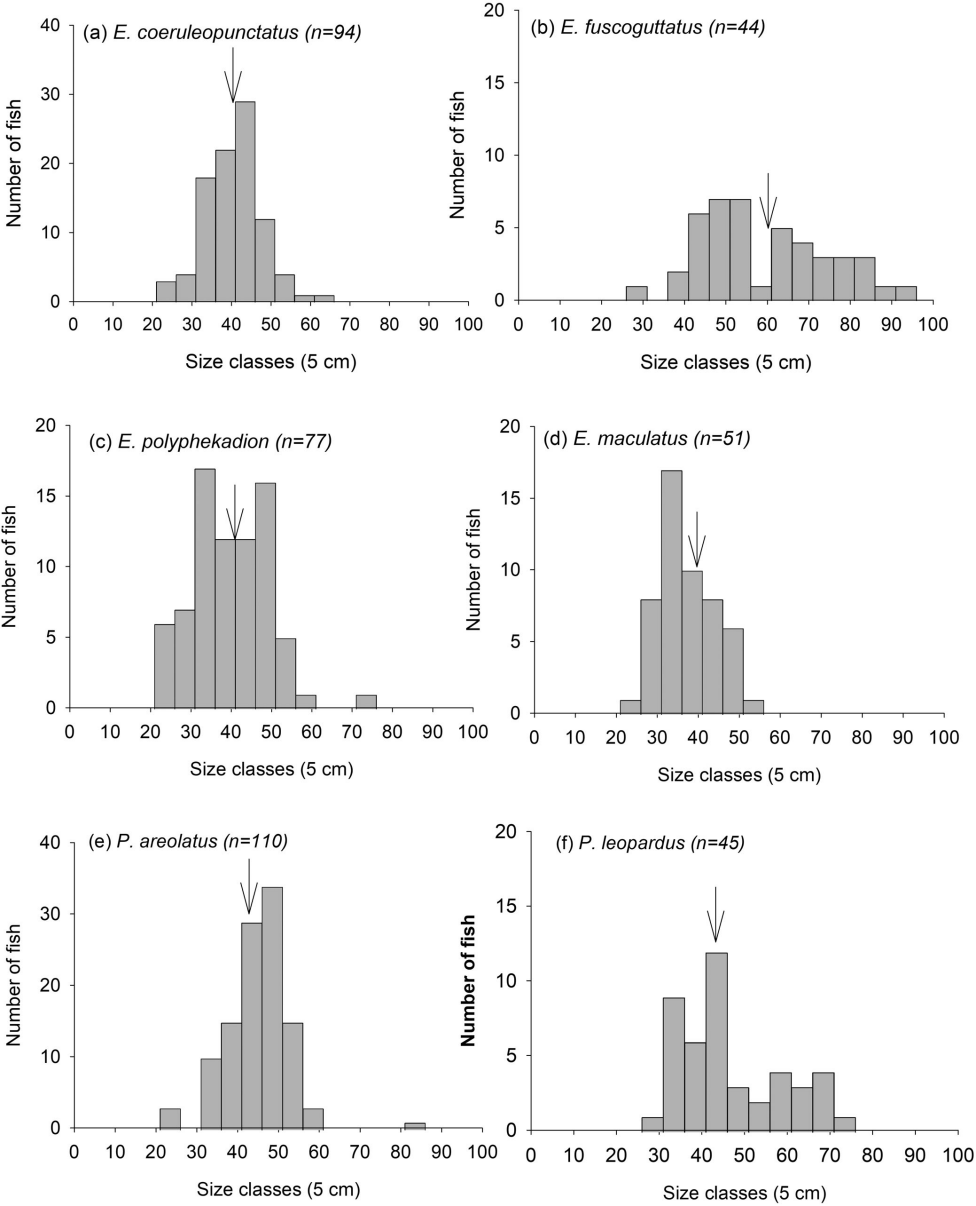
Size class (fork length) distribution of six grouper species at municipal fish markets in Fiji (a) *E. coeruleopunctatus,* (b) *E. fuscoguttatus,* (c) *E. polyphekadion,* (d) *E. maculatus,* (e) *Plectropomus areolatus,* (f) *P. leopardus*. Arrows indicate the 50% size at maturity (L_50_, Prince et al., 2018). Sample sizes are indicated for each species in brackets. Data are pooled for the years 2016–2018. Source: Biospherics, Ministry of Fisheries, Wildlife Conservation Society, World Wide Fund for Nature. Minimum legal size for groupers is 25 cm total length (Fisheries Act Cap 158, appended); smaller fish are underrepresented in the graphs and are usually consumed by fishers and their households.

Market surveys conducted between 2016 and 2018 found that a high percentage of groupers were sold in municipal markets below the size of maturity (Figure 2). Proportions of immature individuals were particularly high in *E. maculatus* (71%), *E. polyphekadion* (55.8%), and *E. fuscoguttatus* (52.3%), and below 50% in *Plectropomus* spp. Size class distributions of the six most abundant species at the market showed few large fish, that is few above 50 cm FL for most fish, with individuals as small as 20–30 cm being sold (Figure 3).

Length‐based assessments showed that six species were fished at about four to five times the level that would maximize their sustainable yields, and that the SPR was below acceptable levels (Prince, 2017; Prince et al., 2019). Without management, catches are set to decline further (Prince, 2017). These findings show that, of all grouper species assessed, all are below the minimum acceptable SPR level of 20% with four species, *E. polyphekadion, P. areolatus, E. maculatus*, and *E. coioides* at seriously low levels of ≤5% SPR (Prince et al., 2018, 2019).

### 4.2 Fisher‐identified trends in key grouper fisheries, aggregation timing, and location

**FIGURE 4:**
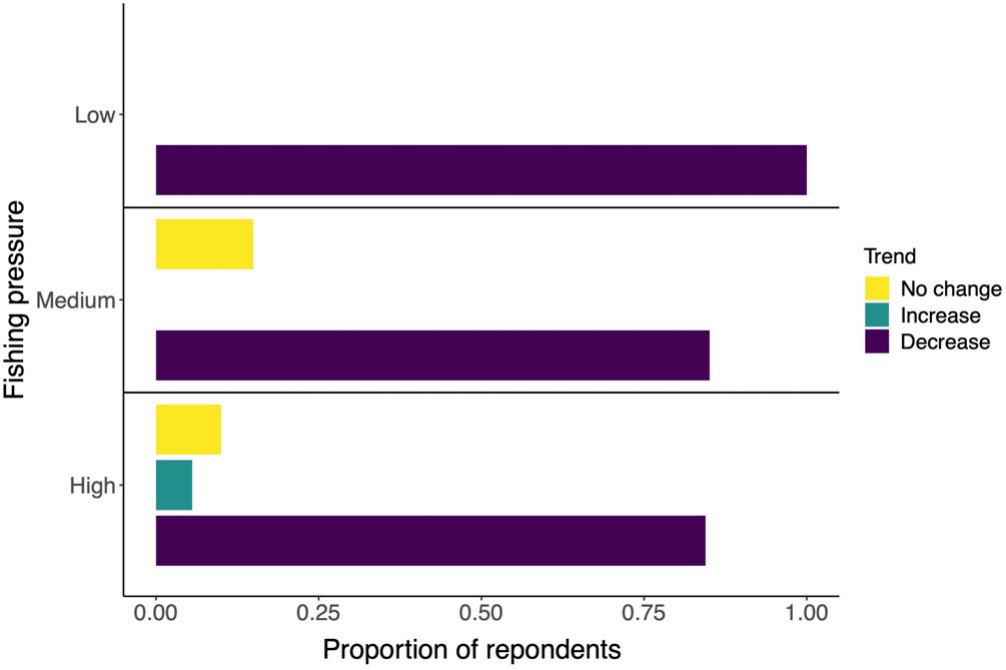
Proportion of fisher interviewees (2003–2005, 2008, 2016, 2017) reporting their personal experiences of general catch trends (catch per trip) of aggregating grouper species across Fiji. Locations are grouped by fishing pressure at the time of the studies: high: Naviti, Viti Levu, and Kadavu = 90 fishers; medium: Vanua Levu = 67 fishers; low: Vanua Balavu and Lakeba = 27 fishers. Total fishers = 184.

**FIGURE 5:**
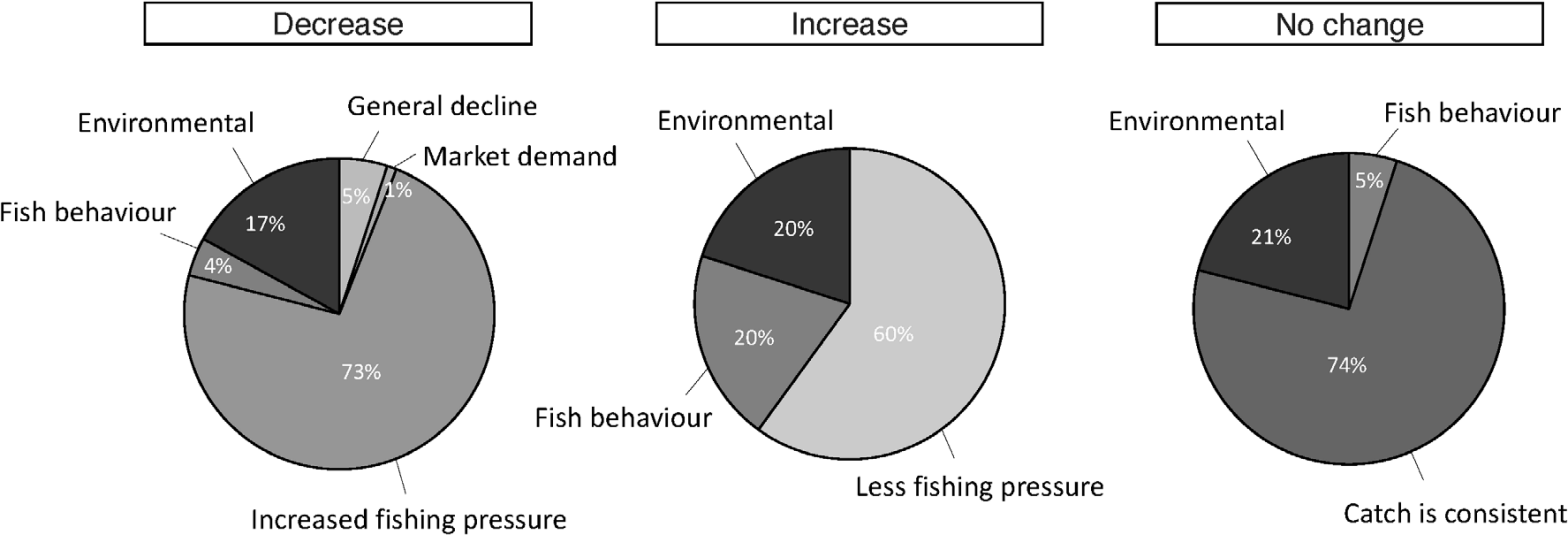
Proportion of fisher respondents from interviews (2003–2005, 2008, 2016, 2017) reporting general trends they observed and perceived reasons for aggregating grouper catch (catch per trip) across Fiji. Decrease = 160, increase = 5, and no change = 19. Total = 184 fishers. “Environmental” refers to perceptions of differences in the weather (18), sea temperature (10), unspecified environment (7), spatial differences of fish without reason (5), and the availability of food/resources for fish (1) between specific sites or over time.

Of 213 fisher interviews conducted 184 provided observations of general catch trends experienced for aggregating groupers (Figure 4). From all areas where interviews were completed most fishers reported a general decrease in catch per trip (*n* = 160, 87%). Only fishers in the high fishing pressure districts reported an increase in catch per trip (*n* = 5; 3%). Across the medium and high fishing pressure districts, 19 fishers (10%) reported no changes in catch/trip over time. The main reason suggested for declines in catch/trip was increased fishing pressure (Figure 5), whereas the main reason given for an increase in catch/trip was low fishing pressure. Other reasons proposed for increased catches included changes in fish behavior, changes in area fished, or attributable to protected areas.

**FIGURE 6:**
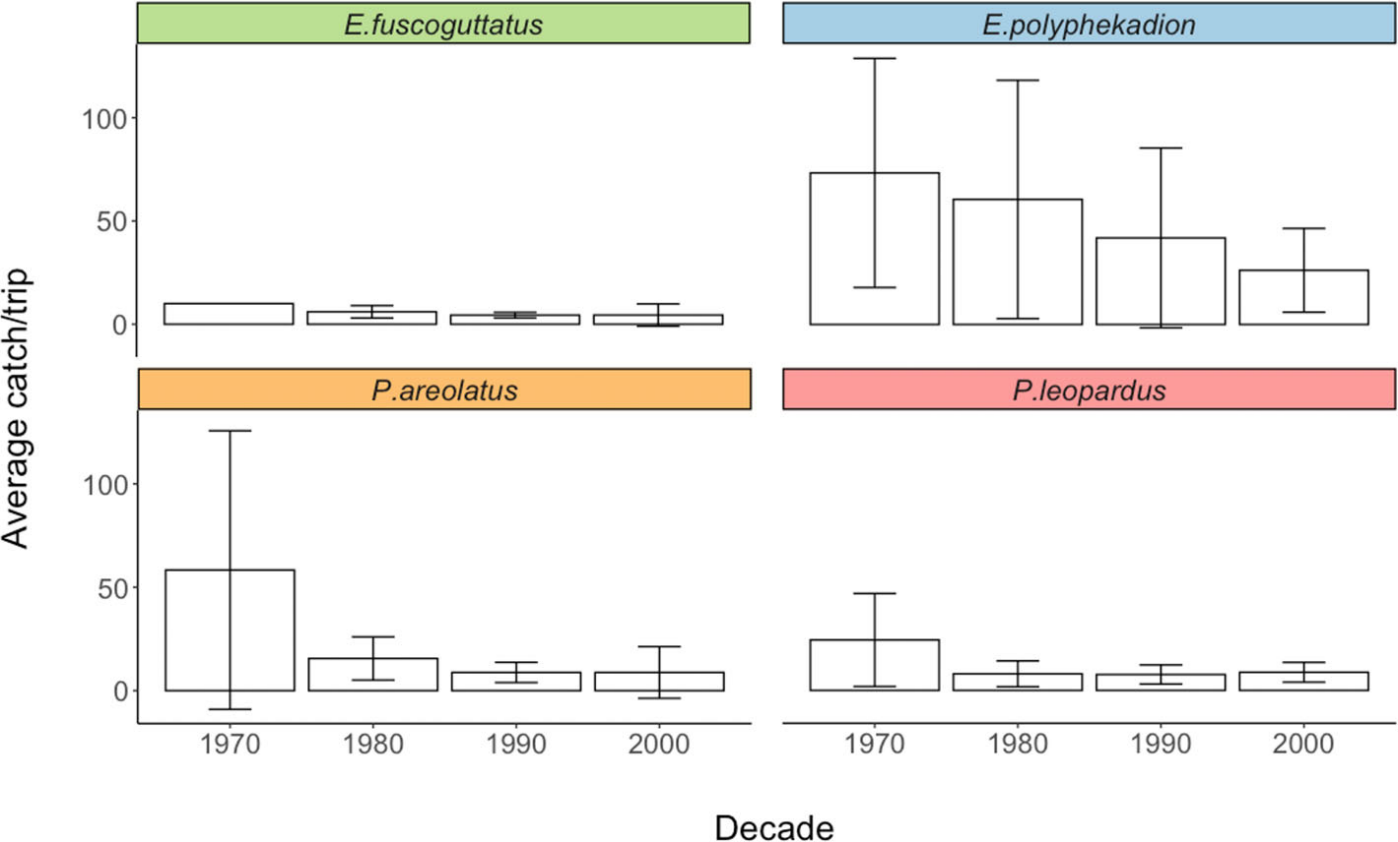
Perceived average catch per trip by decade of the four most common aggregating groupers across Fiji (*Epinephelus fuscoguttatus, Epinephelus polyphekadion, Plectropomus leopardus*, and *Plectropomus areolatus*) as reported by 142 fishers in interviews conducted during 2003–2005 and 2008. SD for each species for each decade is shown.

**FIGURE 7:**
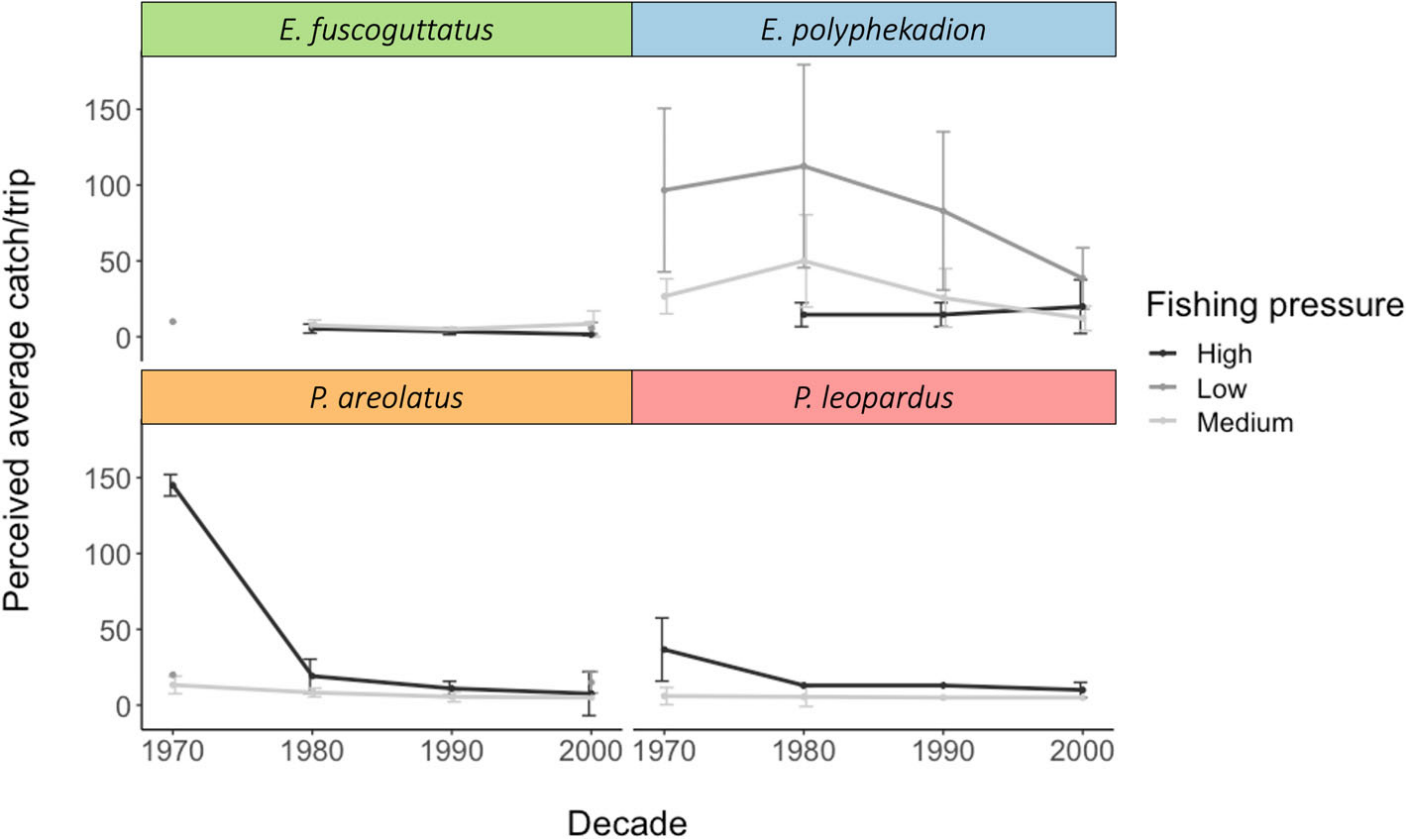
Fisher perceptions of their average catch per trip over time for aggregating groupers, *Epinephelus polyphekadion, Plectropomus areolatus, Plectropomus leopardus*, and *Epinephelus fuscoguttatus*, based on the assigned levels of fishing pressure. High fishing pressure = Naviti, Viti Levu, and Kadavu; medium fishing pressure = Vanua Levu; low fishing pressure = Vanua Balav and Lakeba. Data from 94 fishers collected during 2003–2005 and 2008 interviews. SD bars are shown.

A total of 142 fishers reported average catch/trip values for the most commonly fished aggregating groupers: *E. fuscoguttatus, E. polyphekadion, P. leopardus*, and *P. areolatus* (Figure 6, Table 1). According to these reports, all species declined over time in catch/trip, with *E. polyphekadion* showing the highest overall average catch/trip across decades and *E. fuscoguttatus* the lowest. *E. polyphekadion* and *P. areola*tus showed the greatest overall declines over the time period. These four aggregating grouper species exhibited different catch trends based on the level of fishing pressure (Figure 7; Table 1).

**TABLE 1:** Ranges of perceived average catch per trip by decade (median value in brackets) for the four most common aggregating groupers across Fiji (*Epinephelus fuscoguttatus, Epinephelus polyphekadion, Plectropomus leopardus,* and *Plectropomus areolatus*), as reported by 142 fishers in interviews (2003–2005, 2008) and based on the assigned level of fishing pressure.

*Epinephelus fuscoguttatus*	Decade			
District fishing pressure	1970s	1980s	1990s	2000s
High	NA	2–10 (5)	2–5 (3.5)	1–3 (1)
Medium	NA	5–10 (7.5)	5–5	2–20 (6)
Low	10	NA	NA	3–10 (4)

*Epinephelus polyphekadion*	Decade			
District fishing pressure	1970s	1980s	1990s	2000s
High	NA	8–30 (12)	8–30 (12)	2–45 (10)
Medium	20–40 (20)	20–100 (40)	2–60 (20)	0–20 (14)
Low	50–200 (85)	40–200 (110)	20–200 (80)	10–80 (35)

*Plectropomus areolatus*	Decade			
District fishing pressure	1970s	1980s	1990s	2000s
High	140–150 (145)	10–40 (17.5)	6–20 (10)	0–40 (2)
Medium	10–20 (10)	5–10 (10)	2–10 (5)	5
Low	20	NA	NA	10–20 (15)

*Plectropomus leopardus*	Decade			
District fishing pressure	1970s	1980s	1990s	2000s
High	20–60 (30)	13	13	5–15 (10)
Medium	2–10 (6)	1–10 (5.5)	5–5	5
Low	NA	NA	NA	NA

*Note*: Median values are shown in parentheses. Where just one number is given, only one average value was reported by the fishers. NA means no averages were reported.

Perceptions over time varied according to different species. For *E. polyphekadion*, all perceived average catch/trips declined over time with districts of low fishing pressure having the highest perceived average catch/trip. The average catch/trip in the low fishing district was significantly different from the other (medium and high) districts (Kruskal–Wallis, *p* < 0.01) with differences between low and high fishing pressure districts being more significant (Dunn's test, *p* < 0.01) than those between low and medium fishing pressure districts (Dunn's test, *p* < 0.05) using post hoc tests. For *P. areolatus*, although there were no significant differences in catch/trip among fishing pressure districts, those of high fishing pressure had the highest perceived average catch/trip overall (Kruskal–Wallis, *p* > 0.05). The catch/trip across all fishing districts generally decreased over time. However, not all time periods were covered by these datasets precluding full analysis. For *E. fuscoguttatus*, trends in perceived average catch/trip generally declined over time in districts with high fishing pressure. However, not all time periods were covered by these datasets precluding full analysis. Overall, there were no significant differences in catch/trip across decades or fishing pressure districts for this species (Kruskal–Wallis, *p* > 0.1). For *P. leopardus*, perceived average catch/trip declined over time and was significantly greater in the high fishing pressure districts compared to the medium fishing pressure districts (Kruskal–Wallis, *p* = 0.05). There were no *P. leopardus* reported from low fishing pressure districts.

**FIGURE 8:**
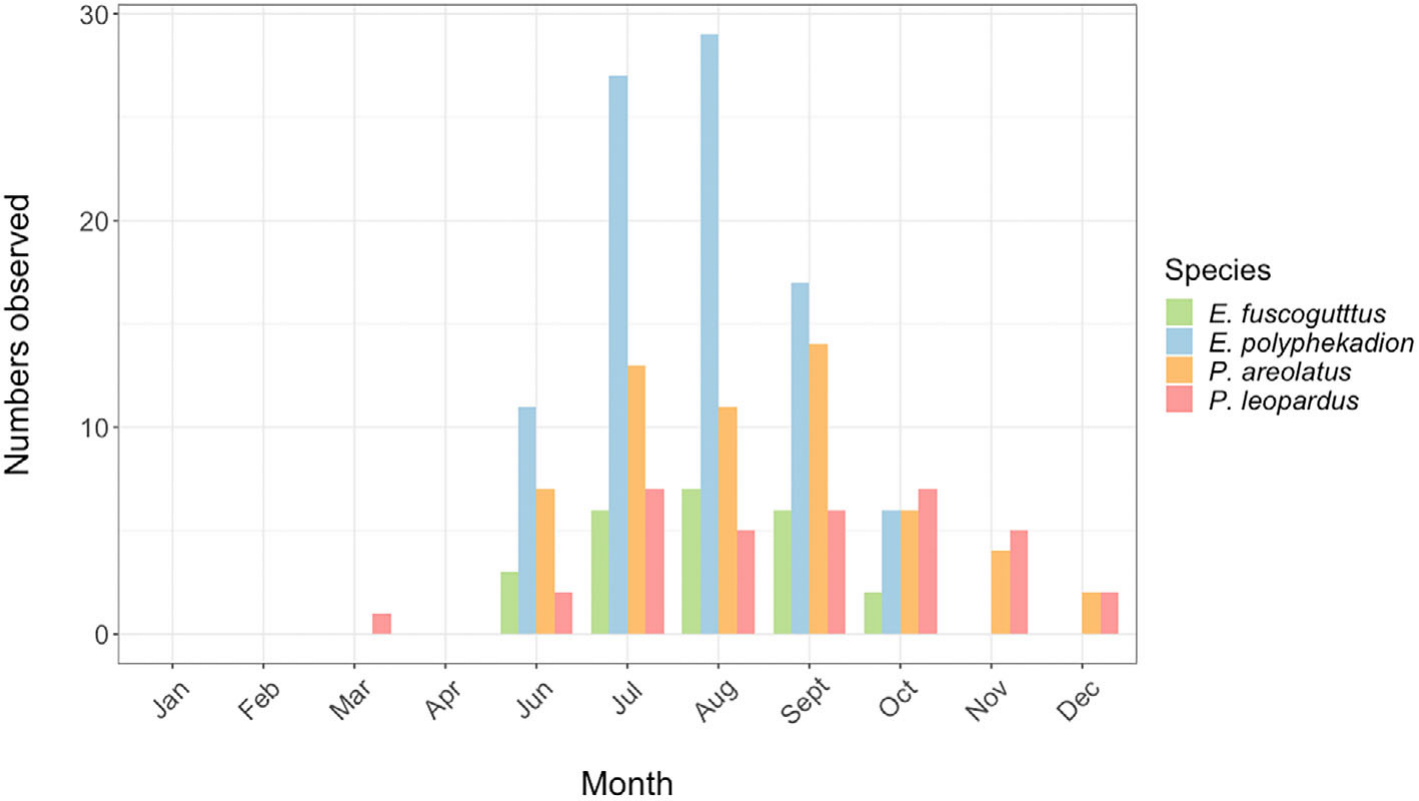
Months identified by fisher interviews (100) during 2003–2005 when groupers aggregate to spawn based on elevated catches and presence of ripe eggs/milt.

Interviews conducted in 2003–2005 explored possible seasonality in elevated landings, fish numbers, and/or fish with eggs, indicative of spawning aggregation formation for the four focal aggregating groupers. Of 79 fishers interviewed reporting seasonality catches were distinctly recalled as being substantially higher than at other times of the year and typically with many fish full of eggs or “milk” (milt) (Figure 8). Many fishers consistently reported that most high catches were taken in the 1–2 weeks leading up to the full moon of their identified season, whereas others could not clearly identify a particular lunar phase. Declines in landings were noted particularly for *E. polyphekadion* and *E. fuscoguttatus*, with complete loss of *P. areolatus* from some aggregation sites from which they had historically been taken.

The results indicated June, July, and August as the main spawning months for *E. polyphekadion*, which had the shortest and most distinctive season based on fishers’ responses (Figure 8). September additionally was identified for *E. fuscoguttatus* and *P. areolatus*. Fishers gave vague responses about when *P. leopardus* had eggs, identifying more months of elevated catches (June–December), compared to the other species and with much variation among fisher responses, even within the same communities, regarding aggregation months. Most indications of seasonality were identified by fishers in low‐ to medium‐fished areas with fishers rarely aware of the existence of any aggregations in heavily fished Viti Levu, including the Yasawas. Grouper fishers who stayed close to shore were generally less aware of grouper aggregations than those who went out to reef edges (where most grouper aggregations naturally occur), although they were familiar with nearshore gatherings of mullet, rabbitfish, and other species.

Aggregation site locations identified were situated around reef passages in outer reef areas adjacent to reef passages or around outer reef promontories, except for *P. leopardus*. Interviews identified about 20 sites reported by at least two fishers, mostly from Vanua Balavu and northern Vanua Levu. Given that interviews covered much of the country where habitat typically associated with aggregation sites is common this is likely a substantial proportion of the total number of exploited spawning aggregation in the country.

Several aggregation sites may no longer host aggregations likely due to overfishing. Mali Passage, located west of Mali Island and within easy reach of the town Labasa, was once an important aggregation site and now appears to be fished out. The same fate is suspected of several reef passages in Bua and Cakaudrove provinces, except where local communities protect them (Fox et al., 2012; Jupiter et al., 2014). Three out of five once‐exploited aggregation sites in Kadavu Province (Namara, Vesi, and Korolevu Passages) were considered by fishers to be no longer viable for fishing.

**FIGURE 9:**
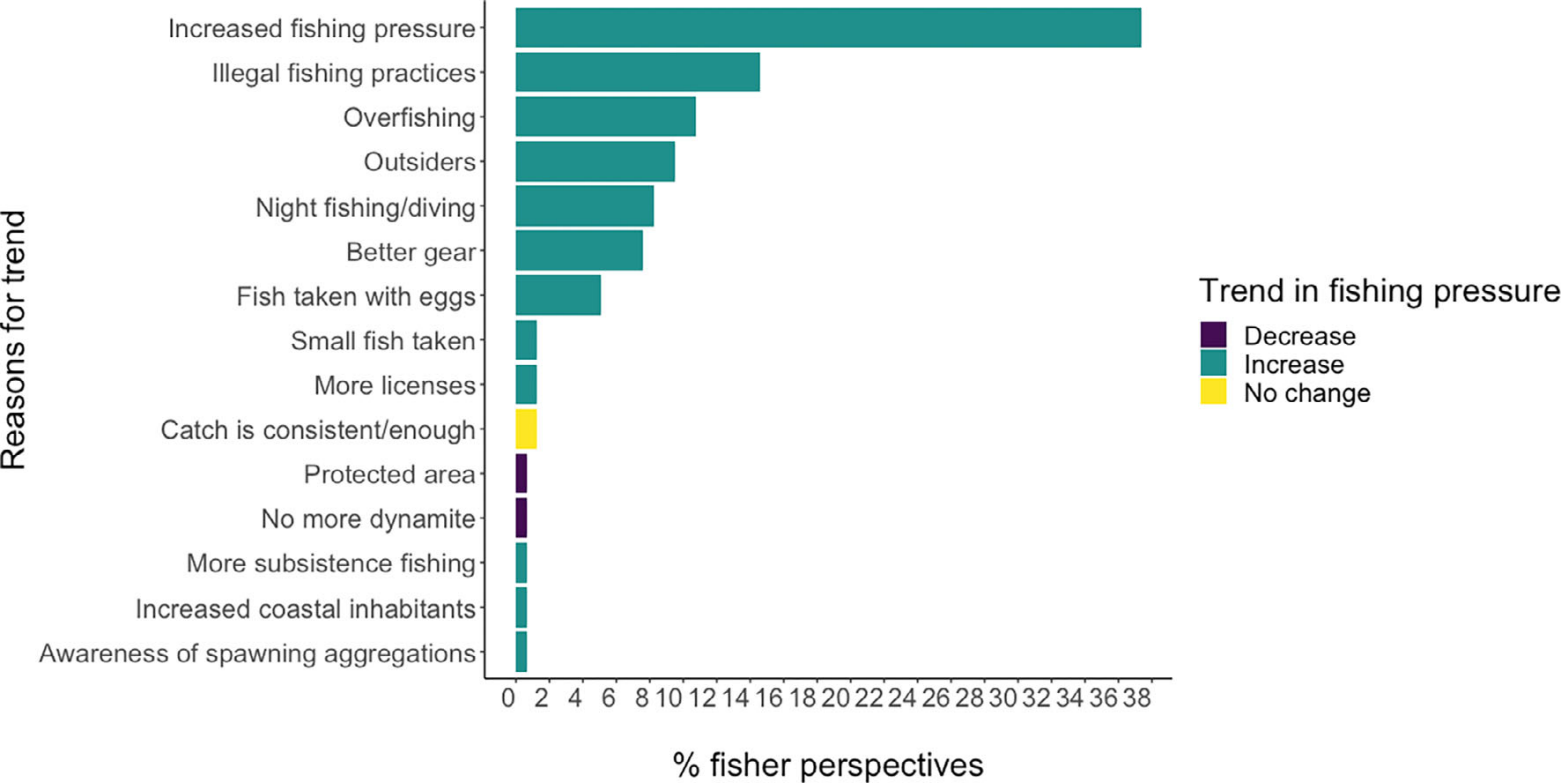
Proportion of fishers interviewed (2003–2005 and 2008) reporting general trends in catches (decrease = 2 fishers, increase = 96 fishers, and no change = 2 fishers) and perceived reasons for reported trends affecting aggregating groupers across Fiji. Time period of trend from the fishers' recent memory: total *n* = 101 fishers and total *n* = 158 reasons (some fishers gave >1 reason).

Fishers reported a general increase in fishing pressure on aggregating groupers over time (Figure 9). Declines noted in grouper catches were mainly attributed to this increase, caused by more boats, increased spear fishing, more fishers (particularly outsiders coming in), more licenses, and more nets. Also identified were illegal fishing practices, better gear, night fishing/diving, increased commercial use, introduction of scuba tanks or compressors by outsider traders (initially used for beche‐de‐mer), and general overfishing. Several fishers noted that aggregations had been more intensively targeted after live fish traders had started operations in the late 1990s.

**FIGURE 10:**
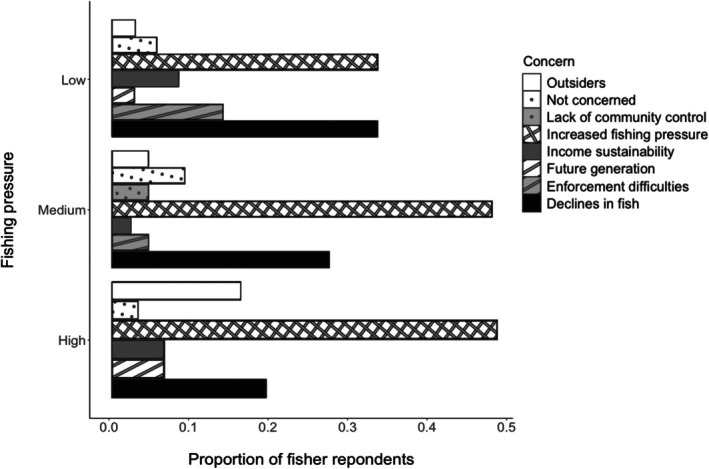
Proportion of fisher interviewees expressing concerns about the state of the grouper fishery across Fiji during 2003–2005 and in 2008. Locations are grouped based on fishing pressure: high: Naviti, Viti Levu, and Kadavu = 31 concerns; medium: Vanua Levu = 44 concerns; low: Vanua Balavu and Lakeba = 36 concerns. Total fishers = 66 and total concerns = 111 (some fishers gave >1 concern).

When fishers were asked whether they had concerns about the aggregating grouper fisheries, and if so what those concerns were, increased fishing pressure and declines in fish were the most common responses (Figure 10). Increased fishing pressure was the greatest concern in high and medium fishing pressure districts and decline in fish was the greatest concern for low fishing pressure. The greatest proportion of concerns about outsiders was noted in the high fishing pressure districts, although fishers from these high fishing pressure districts did not indicate difficulties in enforcement. However, enforcement and lack of control were regularly reported, including the difficulty in some communities to refuse license requests or to repel outsiders. Some fishers noted that fish were getting smaller and thought that size controls were not being enforced.

### 4.3 Underwater surveys of Naiqoro Passage aggregation site

Underwater surveys were conducted from 2008 to 2012 in Naiqoro Passage, northeastern Kadavu. Prior to the first underwater surveys eight interviews with fishers from Matasawalevu, who fished the site, identified seasonally large numbers of *E. polyphekadion, P. areolatus*, and *E. fuscoguttatus*, whereas *P. laevis* reportedly occurred in small numbers; specific moon phases were not indicated. Information on catch rates and the timing of the aggregation season suggested July–September (Kuridrani, 2008; this study). All local fishers interviewed noted declines in catch rates taken in the early 2000s compared to 10–20 years before. Of the three main species indicated reported catch rates per person per day were highest for *E. polyphekadion* and lowest for *E. fuscoguttatus* with *P. areolatus* reported from shallower water than the other two species and its catches having declined substantially.

**FIGURE 11:**
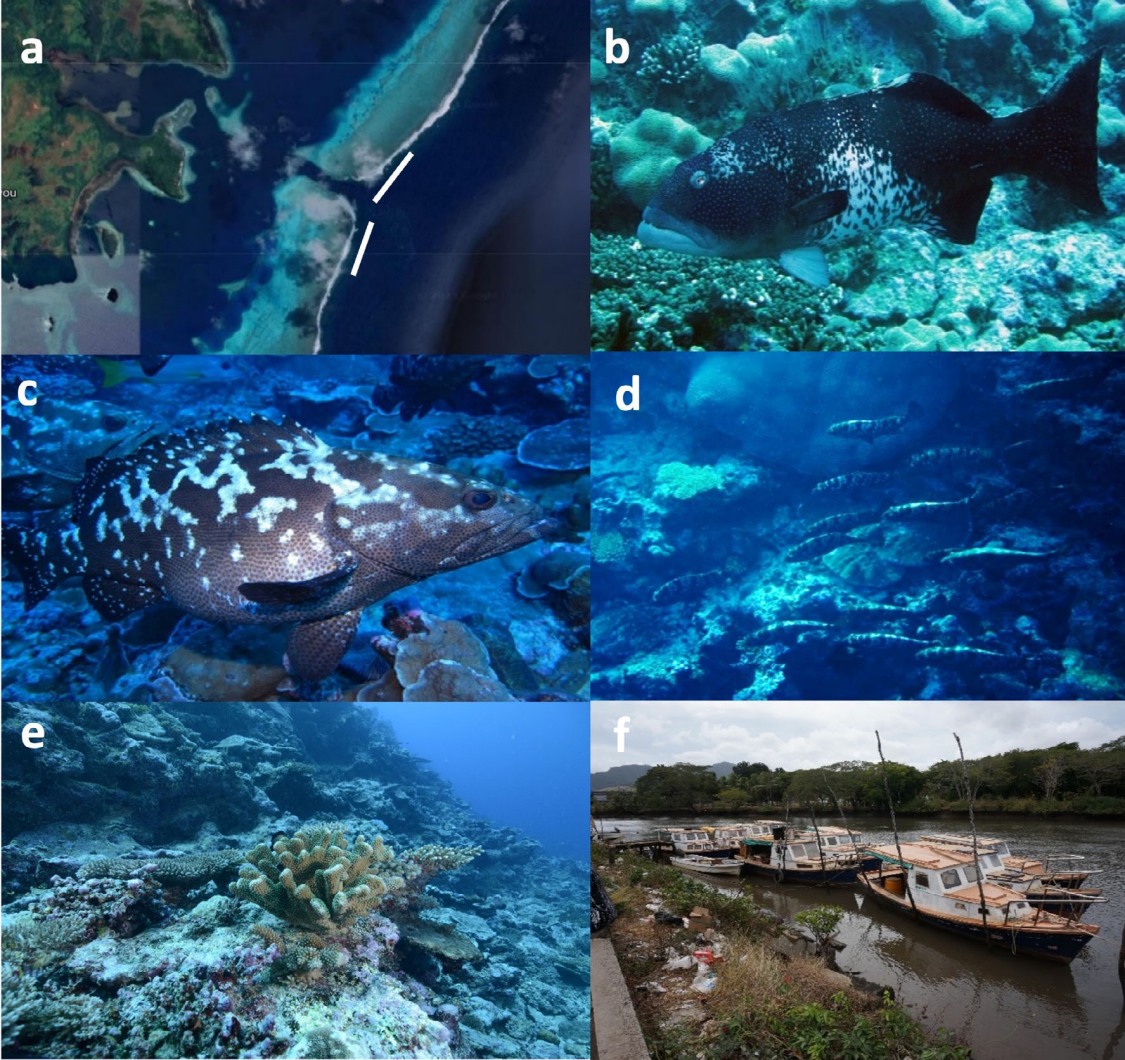
(a) Naiqoro Passage between lagoon and ocean to east of Kadavu Island (satellite image); two white lines show approximate location of aggregation; the survey site extended from the outer channel entrance to S19 00.816 and E178 30.166 to the north, and to S19 00.990 and E178 30.125 to the south. (b) *Plectropomus laevis* putative male in courting color (photo: Stanley Shea). (c) Gravid female *Epinephelus polyphekadion* (photo: Stanley Shea). (d) Migrating *Epinephelus polyphekadion* moving into the lagoon through Naiqoro Passage at end of spawning season in 2011 (photo: Stanley Shea). (e) Seaward reef wall outside Naiqoro Passage. (f) Boats that exploit grouper aggregations in northern Vanua Levu anchored in Labasa 2015; at least 20 larger boats of 32′ with a capacity of 500–1000 kg per trip run multiple trips per spawning season (Sadovy de Mitcheson & Ramoica, 2015) (photo: Yvonne Sadovy)

In 2008, pilot underwater surveys were conducted to establish a sampling protocol. The majority (>50%) of grouper individuals observed were *E. polyphekadion*, with smaller numbers of *E. fuscoguttatus, P. laevis*, and *P. areolatus*, as well as the occasional sightings of *P. leopardus*. Fish were observed between the depth range of 10 and 30 m with few seen at greater depths. Aggregated fish extended over an area both north and south of the seaward mouth of the Passage (Figure 11a). The habitat included extensive hard and soft coral largely on sloping habitat bordering the open ocean (Figure 11b–e). Day‐to‐day fluctuations in fish numbers may not be meaningful because water conditions can influence counts; for example, fish sheltering in stronger current conditions made counting more challenging.

**FIGURE 12:**
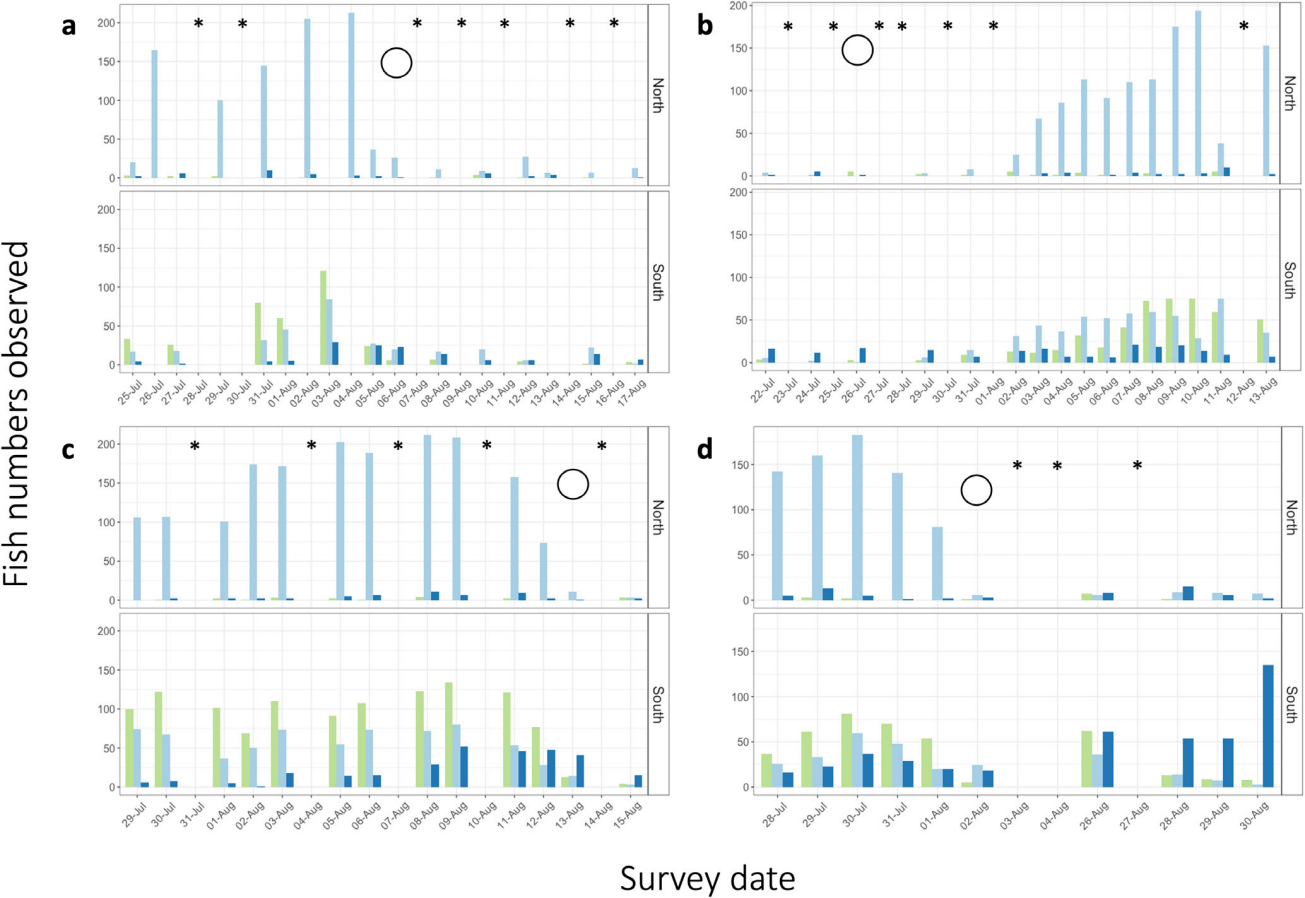
Numbers of *Epinephelus fuscoguttatus* (green*), Epinephelus polyphekadion* (light blue), and *Plectropomus laevis* (dark blue) at Naiqoro Passage counted during surveys of (a) July 25–August 17, 2009; (b) July 22–August 13, 2010; (c) July 29–August 15, 2011; and (d) July 28–August 4, 2012. Daily maximum counts are given separately for the north and south seaward sides of the passage and outer apron for three species. Days with zero counts (*) for all three species were not surveyed. Full moon (open circle) (a) August 6, 2009, (b) July 26, 2010, (c) August 13, 2011, (d) August 2 and 31, 2012.

In 2009, 15 underwater surveys were conducted between July 25 and August 17 (full moon August 6). Three grouper species were regularly recorded: *E. polyphekadion, E. fuscoguttatus*, and *P. laevis*. For *E. polyphekadion* and *E. fuscoguttatus*, numbers increased until the full moon following which most departed the spawning site (Figure 12a). For *P. laevis* numbers started to increase closer to, and stayed elevated a little beyond, the full moon.

Spatially, *E. polyphekadion* predominated to the north of the passage entrance and *E. fuscoguttatus* and *P. laevis* to the south. Maximum daily numbers recorded were 213 for *E. polyphekadion*, 121 for *E. fuscoguttatus*, and 29 for *P. laevis*. *E. fuscoguttatus* became extremely active after surveys began, females were heavily gravid, and pair‐spawning was observed during August 2 and 3 (5.40 p.m.). *P. laevis* exhibited intense activity with dark‐colored putative males (Figure 11b) patrolling several meters above the substrate and clusters of 9–10 fish (possible females) seen at about 30 m over a sandy flat. As *E. polyphekadion* numbers built fish were increasingly active with chasing, changing colors, courting, and gravid females (Figure 11c) evident.

In 2010, 16 underwater surveys were conducted between July 22 and August 13 (full moon July 26), with a single diver doing spot‐checks in the second half of August. Three grouper species were regularly recorded; *E. polyphekadion, E. fuscoguttatus*, and *P. laevis*. Spatially, *E. polyphekadion* predominated to the north of the passage entrance and *E. fuscoguttatus* and *P. laevis* to the south. Maximum daily numbers recorded were 230 *E. polyphekadion*, 76 *E. fuscoguttatus*, and 25 *P. laevis* (Figure 12b). Fish numbers started to increase about a week after the full moon in late July, to the August new moon. Although full surveys finished on August 13, with numbers still elevated at that time, a single experienced diver visited the site to follow up during August 17, 24, and September 2. We noted that the number of fish remained somewhat elevated and actively chasing until around August 24 after which it dropped by early September when few fish were left on‐site.

Observations of fish numbers, colors and behaviors suggested that the aggregation had started to build about a week after the late July full moon terminating around the time of the full moon in late August (August 24), with some reproductive activity indicated around the mid‐August new moon. Large *P. laevis* were observed chasing throughout much of the survey period with the larger fish displaying white bellies and defending areas. *E. fuscoguttatus* gravid females occurred around the new moon in August, with fish very actively chasing. *E. polyphekadion* showed much chasing and jaw‐to‐jaw fighting around the new moon with gravid fish increasing from early August and color changes observed. *P. laevis* occurred in small groups of putative females and a single putative male; dark body with white area midventrally, white rings around the eye,and white lips (Figure 11b) displaying agonistic behavior in the form of shaking head and rolling sideways. The largest fish in the group often “patrolled” high up in the water column and aggressively chased away similar fish.

In 2011, 13 underwater surveys were conducted from July 29 to August 15 (full moon August 13). Three grouper species were regularly recorded: *E. polyphekadion, E. fuscoguttatus*, and *P. laevis*. Few *P. leopardus* were spotted. Spatially, *E. polyphekadion* predominated to the north of the passage entrance and *E. fuscoguttatus* and *P. leopardus* to the south. Maximum daily numbers recorded were 288 *E. polyphekadion*, 134 *E. fuscoguttatus*, and 59 *P. laevis* (Figure 12c). All three species peaked a few days before the mid‐August full moon quickly declining thereafter. Gravid *E. polyphekadion* were evident shortly before the full moon (Figure 12c). A small school of camouflage grouper moving into the lagoon through the passage just after spawning terminated was seen (Figure 11d); this behavior had not been documented before in Fiji but a local dive guide had seen similar such large schools in the past. For *P. laevis* intense activity was observed, with putative males patrolling high up in the water column and across a deeper sand flat, and groups of up to 10 smaller fish were seen a little deeper over a sandy flat.

In 2012, 11 full underwater surveys and additional brief surveys were conducted between July 28 and August 4 (full moon August 2 and 31), and during August 26 and 28–30; dives were not possible in the intervening period. As for previous years three grouper species were regularly recorded: *E. polyphekadion, E. fuscoguttatus*, and *P. laevis*. Few *P. leopardus* or *P. areolatus* were noted. Maximum daily numbers recorded were 243 *E. polyphekadion,* 83 *E. fuscoguttatus*, and 137 *P. laevis* (Figure 12d). Fish numbers built toward the early August full moon with chasing and color changes with *E. polyphekadion* and *E. fuscoguttatus* dropping at the full moon with a slow decline in numbers thereafter. *P. laevis*, increased between the two August full moons peaking just prior to that in late August. This species exhibited several color forms and some fish were gravid.

In summary, underwater surveys at Naiqoro Passage were conducted annually in July and August from 2009 to 2012 during all phases of the lunar cycle. Few fishes were present on‐site, both before and after the short aggregating periods, and the regular presence of gravid females and behaviors (courting and chasing), and color changes observed elsewhere, were associated with the spawning season. Although the area was not surveyed in other months due to logistical constraints regular local tourist dive operations at the site concurred that those few (Southern Hemisphere) winter months were aggregation months for groupers at Naiqoro.

In terms of species, *E. polyphekadion* and *E. fuscoguttatus* matched fisher responses for aggregating grouper species present but fishers had also identified large numbers of *P. areolatus* caught in shallow waters in the past, which our surveys did not record. *P. laevis* was only occasionally reported by fishers (as local name *donu loa*) but because fishers readily distinguish between the two *Plectropomus* species we do not consider that these were confused in interviews. In all years *P. areolatus* was rarely documented and always observed in the shallowest areas; from these observations, combined with interviews, we conclude it likely that *P. areolatus* was once abundant and, being the shallowest species of those considered, may be particularly susceptible to spear fishers (e.g., Hamilton et al., 2012).

## 5 DISCUSSION

### 5.1 Importance of groupers and status of grouper fisheries

Groupers are an important, heavily targeted, and increasingly compromised taxon in the coastal fisheries and economies of the Pacific. Fiji is no exception. Although information and data from multiple and diverse sources compiled for this evaluation are of varying quality, overall conclusions paint a consistent and worrying picture. Estimated annual landings of groupers increased at least tenfold in the past three decades, from hundreds of tonnes in the 1990s to well over 1000 mt in recent years and make up 5%–12% of total coastal fish landings, in line with much of the Pacific. Annual grouper exports appear to be less than 100 mt, highlighting the importance of the taxon domestically where it is increasingly in short supply according to traders.

Among grouper landings 25–30 species are taken mainly from four genera; *Epinephelus*, *Plectropomus*, *Cephalopholis*, and *Variola* (Lee et al., 2020). Many of the larger species for which there are data are caught and sold while still in their juvenile size range. Among the most highly valued groupers in trade, due to both volumes landed and market value, are several aggregation‐spawners, notably *E. polyphekadion, E. fuscoguttatus, P. areolatus*, and *P. leopardus*. These four species are the major focus of this study because of all groupers they are among the best understood, based on this review of available information, collectively the most valuable, and are particularly susceptible to overfishing due to their aggregating habit.

Clear seasonal aggregation patterns of several months annually were identified by fishers for each of *E. polyphekadion*, *E. fuscoguttatus*, and *P. areolatus* but were less clear‐cut for *P. leopardus*. This latter species differs in its aggregating behavior from the other three, which tend to form large aggregations at a few sites over a distinct few months each year. *P. leopardus*, by contrast, forms multiple scattered small aggregations rather than a few large ones (Samoilys, 1997). Moreover, unlike the other three species, *P. leopardus* is extensively caught outside of the aggregation season. Such factors would make aggregation seasons and locations less easy to detect for this, compared to the other three, grouper species in Fiji. *P. leopardus* with eggs may be found well into November, therefore appears to aggregate for more months than the other three species (A. Batibasaga, personal communication November 11, 2024).

Of fishers interviewed 87% reported decreases in catch per trip for groupers especially after the 1980s. Although not all trends were statistically significant overall patterns were consistent. Fishers attributed declines in catch rates to increased fishing pressure, more fishers, and more gears. Also noted were declining aggregation catches from many spawning sites. Sites off northern Vanua Levu, in particular, have been increasingly targeted over the past two decades with boats staying out fishing for multiple days until their freezers are filled (Figure 11f) (Sadovy, 2006; Sadovy de Mitcheson & Ramoica, 2015). According to fisher responses aggregations were not evident at all in heavily fished western Fiji at the time of interviews, such as in Lomaiviti and the Yasawa Island groups,  where there is habitat suitable for aggregation sites, suggesting their likely disappearance. Three out of five known aggregations around Kadavu were either reduced or no longer viable. A once‐important aggregation at Mali Passage has disappeared completely (Sadovy, 2006). These concerns are not new. In the late 1990s and early 2000s, exports of live groupers exceeded levels considered to be sustainable and spawning aggregations were targeted heavily; concerns led to cessation of this export fishery (Ovasisi, 2006; Sadovy, 2006; Teh et al., 2009; Yeeting et al., 2001).

Although most individual fishers experienced declines in grouper catch rates in their regular fishing grounds over time (increases reported were mainly associated with specific targeting of *P. leopardus* in recent years), overall catches at the national level increased. The increase in national catches is associated with growing efforts to locate new aggregations, expansion of fishing grounds, and greater interest to source groupers, including for export. It is also attributable to increased fishing pressure, overall, as reflected in the increases in coastal fishing licenses issued between 1978 and 2022 (Supplementary Information S2). The finding that localized depletions in catch rates (per fisher trip or in specific communities) are not detectable at the national (consolidated catches) level obscures the overall degradation in grouper stocks which is only otherwise detectable through independent studies, such as the SPR analyses, market surveys, and so forth.

### 5.2 Safeguarding Fiji's valuable grouper fisheries

Recognizing the importance of groupers in the economies of many tropical island countries, the increasingly heavy targeting of the taxon, serious declines in many grouper fisheries, and the number of species assessed as threatened due to overfishing, better management is clearly needed to safeguard the economic and food security benefits that these bring to communities. Concerns are particularly acute for aggregating species. A growing number of countries (including Australia, Fiji, Papua New Guinea, Seychelles, Solomon Islands, and the Federated States of Micronesia) in the Indo‐Pacific have acknowledged and variously responded to this need (e.g., Grüss et al., 2014; Hughes et al., 2020; Rhodes et al., 2021; Robinson et al., 2015; Robinson & Samoilys, 2013; Sadovy de Mitcheson & Colin, 2012).

Importantly and encouragingly, success stories show positive outcomes for fish numbers and fisheries, when protective measures are developed based on good science and effectively implemented and enforced (e.g., Hamilton et al., 2011; Nemeth, 2005; Sadovy de Mitcheson, Linardich, et al., 2020; Waterhouse et al., 2020). However, protection is needed before numbers decline too markedly; once an aggregation drops to below threshold numbers recovery might be compromised due to depensation (this occurs when per capita population growth in numbers decreases at low population densities). Hence, protection is needed before, not after, aggregation numbers become severely reduced; that is, precautionary management is needed for aggregation fisheries (e.g., Sadovy de Mitcheson, 2016; Sadovy & Domeier, 2005; Waterhouse et al., 2020).

In Fiji there are multiple indications that groupers need stronger and more effective management than is currently in place. Although there are no provisions within the current Fiji Fisheries Act that provide for legal controls on fishing aggregations (Fox et al., 2012), several measures directly or indirectly control their exploitation, protect their sites, or otherwise aim to manage the taxon. These include seasonal, spatial, minimum size, fishing, and gear control measures (Supplementary Information S3) and represent an excellent foundation from which to build stronger and more effective protection.

In 2018, the Ministry of Fisheries introduced the first 4‐month annual spawning season moratorium on harvesting 27 grouper species from June 1 to September 30, 2018. This measure bans harvest, sale, purchase, possession, or export of these species during the 4‐month closure. Although this seasonal moratorium, against scientific advice, was partially lifted from August 1, 2020 (i.e., reduced to only 2 months) because of the COVID‐19 restrictions and economic downturn, and again in 2021 and 2022, the annual 4‐month seasonal spawning aggregation was back in place in 2023. Offenders can have their fish confiscated and receive high fines of USD4424–22,123 for individuals and USD8849–44,247 for corporations or businesses. The level of the fine depends on the severity of the offence as determined by the Fijian Court System. Since 2017 penalties of between USD869 and 8695 per infringement have been applied to several individuals and to a business company.

Spatial protection, if well implemented and appropriately located, can be effective for managing reef‐associated fishes by safeguarding key habitats such as nursery areas, spawning sites and reefs. In 2018 Fiji gazetted the Naiqoro Passage Spawning Aggregation Marine Reserve as the first in the country to specifically encompass an aggregation site. There is also a wide network of locally managed marine areas (LMMAs) including traditionally managed, or *tabu*, areas that might offer some protection to groupers such that community‐based management can help safeguard certain aggregating groupers (Veitayaki, 1998). Although most *tabu* areas are small relative to the movement patterns of medium‐to‐large‐sized groupers and do not explicitly include aggregation sites there are exceptions (Goetze et al., 2016). The Namena Marine Reserve (88 km^2^) and Vatu‐i‐Ra Conservation Park (110 km^2^) are probably large enough to protect some grouper species for a significant part of their life cycle. However, the extent to which they encompass grouper aggregations or other key habitats for the taxon is unknown. In Bua Province, Kubulau District, communities banned grouper catch during August and included one identified spawning site in a *tabu* area where commercial fishing was banned (Clarke & Jupiter, 2010; Fox et al., 2012). The Fiji government is working on establishing networks of connected locally managed marine areas under a new national marine protected area (MPA) Ledger and Regulation programme for early 2025.

Licensing controls are, in principle, possible. Fijian law recognizes 410 inshore traditional fishing grounds (i‐Qoliqoli) associated with 850 Indigenous Fijian (i‐Taukei) communities (Prince et al., 2020) which can exercise some control over their waters. However, in practice their ability to manage their own fisheries is limited. Although the current Inside Demarcated Area (IDA) licensing system gives the i‐Taukei chiefs some control and management oversight of who is permitted to fish and trade catch from their i‐Qoliqoli area (Mangubhai et al., 2019), interviewees suggested that communities felt little empowered to control, although there are exceptions (Fox et al., 2012). Licenses for fishing and exports should be approved only if there is sufficient underlying resource base to support such activities; currently, this is not a consideration.

Gear and size restrictions can be effective input and output management approaches. For example, scuba and hookah are prohibited for fishing which, if enforced, could substantially protect groupers in aggregations where scuba is often used; night‐time spearfishing with flashlights is having a major impact on parrotfish and grouper spawning aggregations in many parts of the Pacific (Gillett & Moy, 2006). Setting minimum legal size limits for species is possible under the Fisheries Act. For groupers this is currently 25 cm for all species, irrespective of their size of sexual maturation. This means that for the 11 species of groupers most frequently sold in municipal seafood markets on Viti Levu and Vanua Levu (major markets) the current legal‐size limit is considerably below the recommended L_50_, the size at which 50% of the population is mature (Prince et al., 2018). Improved measures could lead to increases in SPR above their currently critically low levels.

Moving forward, there are two key management recommendations from this study: (1) improved spatial and temporal management during the spawning season and (2) revision of minimum sizes for capture and sale. Further research is needed to fine‐tune the duration (starting and end months) of seasonal protection for focal species. More aggregation sites could be gazetted for spatial protection or incorporated into MPAs or LMMAs. Revised and enforced minimum capture and sales sizes could better protect juveniles of key species of concern; in most cases this means an increase in the minimum size, quite substantially in the case of larger species (Prince et al., 2018).

Complementary measures to inform management, support enforcement, and assess outcomes include regular data collection on grouper catches, use and trade (both domestic and export), and fishery‐independent field studies, such as surveys at selected aggregation sites. Targeted enforcement (i.e., limited to the protected season and specific sites and for sizes of fish) could be cost‐effective and could strengthen management. Subsistence use is poorly known for all coastal resources in Fiji (Gillett et al., 2014; Lee et al., 2020; Starkhouse, 2009; Teh et al., 2009), including for groupers and despite its importance for local communities; subsistence use accounts for two‐thirds of coastal landings (Ministry of Fisheries, 2014; Starkhouse, 2009). Exports are sparsely and incompletely documented for reef fishes exported as food, including groupers, with economic benefits to the country unknown.

Finally, management measures can be supported by well‐designed outreach campaigns and educational initiatives. The 4FJ Fish Smart campaign builds on earlier momentum to promote the national protection of groupers in their peak spawning season from June 1 to September 30 (https://4fjmovement.org). This campaign was initiated by SeaWeb in 2012 (Kate, 2012) on the back of some of the work reported in this paper and galvanized political and public support to implement a seasonal ban on the harvesting, selling, or storing of groupers in the four peak spawning months. It led to a broad understanding of the need for protection. In partnership with c‐Change, the campaign worked with many sectors of society which collectively created a highly focused and successful public awareness initiative that continues today. The campaign not only highlighted the importance of grouper reproduction for the fisheries but also attracted public attention to the role of individual actions for ensuring that these valuable species are properly managed and can thrive into the future (Ministry of Fisheries Annual Report, 2020–2021, 2018–2019).

An improved understanding of grouper fisheries and their history highlights the high economic value of this taxon to countries and emphasizes the importance of the key management measures of improved spatial and temporal aggregation protection and increased minimum capture sizes. Given the realities of limited funding and capacity typical of most fisheries departments in the Pacific, collaboration and trust should be built with other stakeholders at the national level. Government should be prepared to integrate and apply new knowledge gained by NGOs and the scientific community and empower local communities to manage their own resources to more effectively counter the increasing pressure to exploit and export groupers. Successful management would contribute substantially toward island nation food security now and into the future, a safeguard currently at substantial risk in many countries in the region (Bell et al., 2009).

## AUTHOR CONTRIBUTIONS

Yvonne Sadovy de Mitcheson: ideas, data generation, funding, and manuscript preparation. Aisake Batibasaga: ideas, data generation, and manuscript preparation. Chloe E.R. Hatten: data analysis and manuscript preparation. Sangeeta Mangubhai: data analysis and manuscript preparation.

## FUNDING INFORMATION

Multiple year funding was provided by the David and Lucile Packard Foundation Grants (2004‐26757, 2007‐31524, 2011‐36432, 2015‐62920).

## CONFLICT OF INTEREST STATEMENT

The authors declare that they have no competing interests or conflicting interests of any kind.

## Supporting information


**Data S1.** Supporting information.

**Figure S1.** Total IDA (Inside Demarcated Area) licenses issued annually between 1978 and 2022.

**Table S1.** Recorded commercial finfish landings (mt) sold across local markets in Fiji, and landings and proportion of landings comprised of groupers, according to government data from 1980 to 2008 (Ministry of Fisheries Annual Reports 2014–2021). The data collection programme ceased after 2008.

## Data Availability

The data that support the findings of this study are available from the authors and from WCS. Restrictions apply to the availability of these data, which were used under license for this study.
