# Supplementary material for: From local knowledge and science to policy: Lessons learned from Fiji's valuable grouper fisheries
Source: J Fish Biol. 2025 Jan 8;107(1):34–51. doi: 10.1111/jfb.16041 (PMC12327161; doi:10.1111/jfb.16041)
Supplement: Supplementary file 1 — Data S1. Supporting information. [file JFB-107-34-s002.docx]

**SUPPLEMENTARY INFORMATION SECTIONS**

**Supplementary Information 1 - Government market data 1980-2008**

Table S1. Total annual volume of coastal artisanal fin-fish landings sold across local markets in Fiji (versus Grouper landing estimates)- from 1980-2008 [as extracted from the annual reports and estimated (in green) by A. Batibasaga]

**
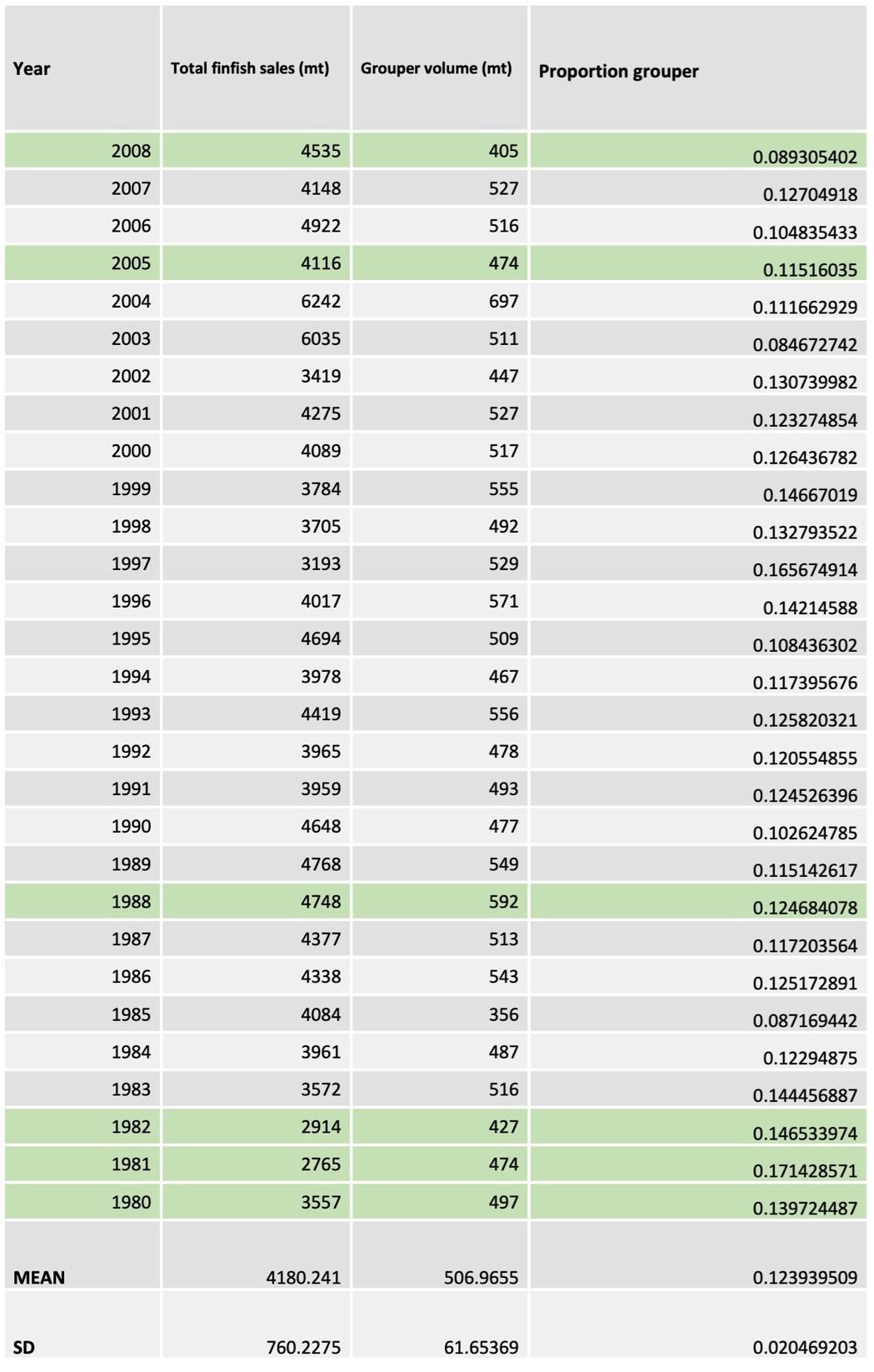
**

**Supplementary Information 2 -IDA LICENCES**

Data from inside demarcated areas (IDA) Licenses (1979-2021) suggest an overall increase in commercial fishing pressure over the last four decades until about 2011, followed by a general decline over the last decade or so (Figure). Licence numbers also reflect events in society (e.g. civil unrest in 1997 and 2000, migration, covid pandemic) and changes in government policy (for example economic input to develop certain areas) that influence license applications and their issuance independently of fishing pressure. Nonetheless, the total number of IDA licenses is understood to be a useful, but minimal, indicator of fishing pressure for two reasons. The first is because they only cover commercial fishing while subsistence (for direct consumption) fishing, which lands approximately two thirds of all coastal fish, does not require a license.

The second is because, overall, license issuance may be typically low in some areas of the country compared to others, irrespective of fisher numbers. For example, in the Eastern Division some fishers sell their catches to an association which takes out a license effectively on their behalf so that the association can market fish; hence a single license may be linked to many commercial fishers none of whom apply individually for a license. Also in the Eastern Division, which is physically far from government offices and oversight, license applications tend to be less in evidence. Central and Western Divisions have traditionally had the highest fishing levels because of urban and market demand pressures for fish and access to transport and the number of licences is highest from these divisions, making up 60% of all licences issued for the 16 years for which data by Division are available (1979-2008 and prior to 2017). More recently, the Northern Division has faced growing market pressure. The data from 2017 onwards should be considered with care since in 2016 the government removed the community payment system linked to the granting of the fishing permit and licence which led to a subsequent reduction in licences issued.

IDA licenses must be consistent with customary fishing rights (i.e., the *i-Qoliqoli*; Fisheries Act Cap 158). They must first be approved by the Customary Fishing Rights Owners (CFROs) and then issued by the government as part of a co-management arrangement between the local *i-Taukei* CFROs and the government. While licenses are typically issued without regard for the capacity of the resource base, CFROs often opt to limit the numbers of licenses issued annually among local community members and outsiders. License data were collected from the Ministry of Fisheries Department annual reports.


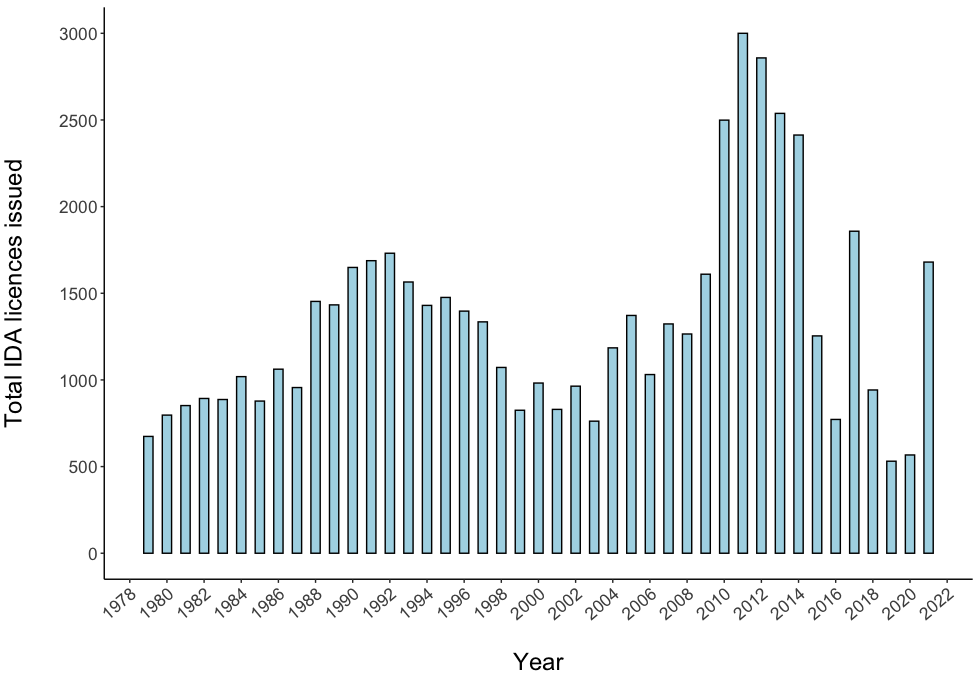


Figure S1. Number of Inside Demarcated Area (IDA) fishing licences issued annually to fish commercially in a coastal or inshore area. Source: Fiji Ministry of Fisheries

**Supplementary Information 3 - Fiji Fisheries Regulations and Policies relevant to Groupers**

| **Year of implementation or most recent amendment** | **Law, regulation or policy and objective(s)** | **Enforcement responsibility & effectiveness** | **Notes** |
| --- | --- | --- | --- |
| Fisheries Act CAP 158 1941; including various amendments since 1945-1991 and other regulations inserted thereafter | Control of undersized fishes and invertebrates, and poaching. Minimum size of 250 mm permitted for groupers (catch and sale). | Responsible authorities are the Fisheries Department and other regulatory agencies such as the Police and Navy, and Honorary Fish Wardens.  Enforcement is currently undertaken by MCS Officers within the Offshore Fisheries and Inshore Fisheries Divisions across municipal markets. Undersized animals are confiscated and destroyed. Live undersize crustaceans are released back to the sea. Multiple confiscations have been made and illegal poachers apprehended during land and sea patrols by Police, often leading to court cases (especially in the last 3-5 years). | The minimum size regulation has been under review since 2017 pending amendment (collaborative work with WWF, WCS, FLMMA, Fisheries and other stakeholders) |
| Spawning Aggregation Seasonal Ban: regulation in force since 2018 (with temporary suspension due to Covid-19): 1 June- 30 September each year. | Regulation was mandated under the Fisheries Act 1941 CAP 148, and the Offshore Fisheries Management Regulation (OFMR) 2014. All fishing (catching) and selling of groupers (both *Epinephelus* and *Plectropomus spp.*) at local markets (local sales) and export to overseas markets are prohibited during the seasonal ban. | Penalties for infringements are *FJ$10,000-20,000 for individual fishers, and for companies from FJ$20,000 and up to FJ$100,000. | In 2020, there was an amendment to shorten the moratorium to 2 months, from 1 Aug-30 Sept. 2020, and the ban was lifted in 2021 and 2022 due to Covid-19. The ban was back in place in 2023 for the full 4 months ban. |
| Fisheries Act, Cap 158, 1941  Seasonal area-based protection  Seasonal protection is for periodic no-take periods, but areas can be opened on certain occasions to support community or church development aspirations | Groupers and other reef fish species are protected in “declared” or community-managed tabus or protected areas. | Supported by the local Fiji Locally Managed Marine Area Network working with local communities across Vanua Levu. Patrols are done by local Fish Wardens and Customary Fishing Rights Owners (CFROs) who have very active *i-Qoliqoli* Committees that do the MCS and Enforcement on the ground 24/7. These are largely done while fishing, as there is no funding to support fuel for patrols. Fuel is either supplied by the Fisheries Department or the *i-Quoliqoli* committee or other stakeholders, such as resorts that use the area. | This is often done through traditional management systems.  Relevant stakeholders include the District and Village Chiefs and Leaders of Local communities, FLMMA, NGOs, other Government Ministries, and Resort Owners and Managers, etc. |
| Legislation:  1. Fisheries Acts, CAP 158, 1941.  2. Offshore Fisheries Regulation 2014. | Scuba and hookah are prohibited for fishing. Prohibited also are destructive fishing methods such as the use of rotenone (Derris) poison fishing and dynamite, etc. | Fines start at $10,000 for individual fishers, and for companies at FJ $20,000, going up to FJ $100,000.  Penalty notices given for each infringement, from $2000, or higher fines, depending on the level of infringement. For example, penalties have been imposed and paid since 2015 for harvest or sale of prohibited/threatened species such as Humphead Maori Wrasse (*Cheilinus undulatus*); penalty notice of $20,000 was applied to some local companies. | Fisheries Act, Cap 158 1942 has very minimal penalties/infringement fees.  OFMR 2014 has higher penalties- which also apply to offshore tuna industry stakeholders, with penalties for individual (FJ $10,000-50,000), and corporate entities (FJ$20,000-100,000). Fixed penalties start at $2,000 -$20,000 per infringement. |
| Gazetted Marine Reserve (Naiqoro Passage Spawning Aggregation Marine Reserve):  Gazetted 1  9 January 2018 (4.83 km²) under section 9 of the Fisheries Act (1941)-Legal Notice No. 4). | This Marine reserve is locally called a ‘gazetted MPA [Marine Protected Area],’ as its creation was by way of the promulgation of specific regulations brought into force by being published in Fiji Government ‘sGazette.  Purpose is to conserve, protect and maintain marine biodiversity in the passage and surrounding demarcated area as well as completely protect the grouper spawning aggregation in that special Reef Passage Spawning Aggregation Area. | Enforcement is considered efficient and cost-effective. Surveillance includes observation points on land where community members can see the passage area and  check for vessels or torches or lights used by fishing boats and illegal fishermen. Available are binoculars, including a night vision camera. Shore-based surveillance reduces the need for fuel with a patrol boat that is only used when a fishing boat or divers are observed to be on or near the reserve or marine protected area.  Enforcement is jointly undertaken by local community members, including Fish Wardens at Matasawalevu village, the Fisheries Department, Police Department, Provincial Office, District Officer, and the FLMMA Kadavu Yaubula. The Enforcement Management Support Team (KYMST) is coordinated jointly by the Fisheries Department and other government agencies (e.g. Provincial Office or Principal Administrator) from Vunisea Office. | Reserve purpose is protection in place  throughout the year for sharks, rays, cetaceans, sea turtles, whales and dolphins and other marine organisms including coral and holothurian species within the demarcated area in addition to protection of the grouper aggregation area |
| 8 January 2018 (gazetted)-Kiuva Marine Reserve (7.3 km²).  Mandated under both the Fisheries Act CAP 158, 1941 and the OFMR 2014. | The regulation aims to conserve, protect and maintain diversity and its productivity and protects fishes, invertebrates, sea turtles, rays, including highly endangered and rare species (such as guitarfishes and sawfishes), whales, dolphins (cetaceans) and corals. Two main reef passages are included. | Enforcement is considered efficient and cost-effective and conducted by Kiuva Village, and adjacent community members and the I-Qoliqoli Management Committee, which is part of the FLMMA Yaubula Management Support Team across the Tailevu Province. Enforcement is coordinated and supported by the Fisheries Department on the ground, and Fish Wardens, survey from the village for illegal fishers on or near the MR at any time (24/7 basis). Fish Wardens take watch at night. Efforts are supported by the Fisheries and Police Departments from Nausori and Wainibokasi Offices.  Several poachers were taken to court in the last 2 years. Any person who continues to contravene or repeatedly contravenes this regulation is liable upon conviction to a fine of not less than $20,000 and not exceeding $100,000 or imprisonment for a term not exceeding 2 years, or both. | The area contains two passages and *E. polyphekadion* and *E. fuscoguttatus* have been fished out since the mid 1980-1990s, as have other reef fishes including Humphead Wrasse. Since 2015 there has been a slow recovery  (Ministry of Fisheries, 2018. Unpublished Report; MRIS Kiuva Management Plan, 2016) |
| Gazetted 20 Feb 2015-Wakaya Marine Reserve (16.4 km²). Mandated by both the Fisheries Act CAP 159, 1941 and the Offshore Fisheries Management Regulation, 2014. | The Regulation permanently protects all reef fish and invertebrate species, including coral, through-out the year, now and into the foreseeable future. It also protects fishes, sea turtles, rays, sharks including highly endangered and rare species (such as guitarfishes and sawfishes), whales and dolphins. | Enforcement across the Wakaya marine reserve is considered efficient and cost-effective. MCS and enforcement efficiency (in terms of cost) and effectiveness (in terms of excluding poachers) is high because the local i-Qoliqoli Management Committee and community members undertake policing and enforcement with no government cost. Enforcement is largely undertaken by the local resort management and staff members, resort-authorised Fish Wardens at Wakaya Island, and supported by the Fisheries Department at Levuka (Ovalau) and Makogai Island, and the Police Department Levuka (Ovalau) | The marine reserve encompasses a number of reef passages and hence protects spawning areas of a range of species. |
| Gazetted 13th August 2014.  Area (1.5 km²).  Shark Reef Marine Reserve (Serua). Principal Act or Legislation is the Fisheries Act, CAP 158 1941; also covered under Regulation 9. | The Regulation has the purpose of conserving, protecting and maintaining shark species and marine organisms including coral within the area completely and permanently protects all coral reef fishes, sharks, and invertebrates throughout the year, now and into the foreseeable future. | Enforcement across the Serua Shark Reef MR is considered effective, efficient and cost-effective. Enforcement is largely undertaken by the local Beqa Adventures Shark Dive Company Management, and the adjoining villages of Galoa and Wainiyabia village, the local schools across the Deuba District, and authorised Fish Wardens. Enforcement efforts are ported by the Fisheries Department at Navua; and the Police Department (Deuba and Navua) | The first reserve to be gazetted in Fiji. Also covers a portion of the reef passage and barrier reef system between Wainiyabia and Galoa Village. The Marine Reserve  also protects individuals of larger fishes, particularly Giant Groupers (*Epinephelus lanceolatus*) and Giant Humphead Maori Wrasses, and other large reef fishes that have become rare such as giant sweetlips, giant trevallies and Black trevallies, among others. |

**FOOTNOTES**

MCS-Monitoring, Control and Surveillance

MPA-Marine Protected Area

FLMMA=Fiji Locally Managed Marine Area network

*Conversion rate FJD to USD on 17.3.2024 (XE Currency conversion was 2.26 FJD to 1 USD

Fish Wardens (=Honorary Fish Wardens) do their work on a voluntary basis, without pay. The duties and responsibilities of the Fish Wardens the same as Fisheries Officers, and other Fisheries Enforcement Officers (such as the Police and Naval Officers).
