# Supplementary figures and images for: From local knowledge and science to policy: Lessons learned from Fiji's valuable grouper fisheries
Source: J Fish Biol. 2025 Jan 8;107(1):34–51. doi: 10.1111/jfb.16041 (PMC12327161; doi:10.1111/jfb.16041)

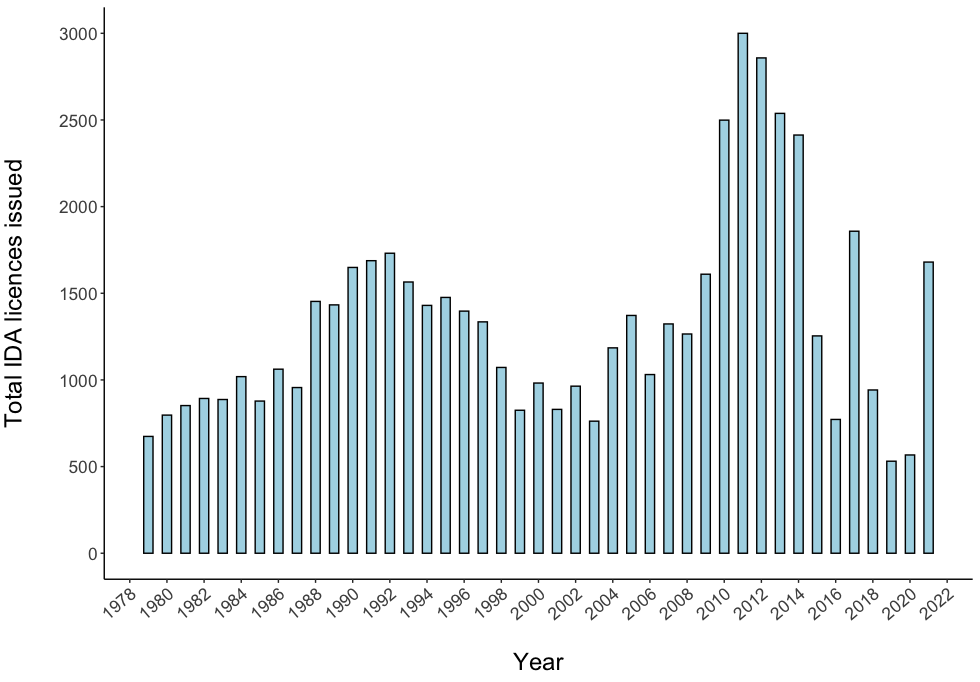

Supplement: Supplementary file 2 — Figure S1. Total IDA (Inside Demarcated Area) licenses issued annually between 1978 and 2022. [file JFB-107-34-s001.tiff]

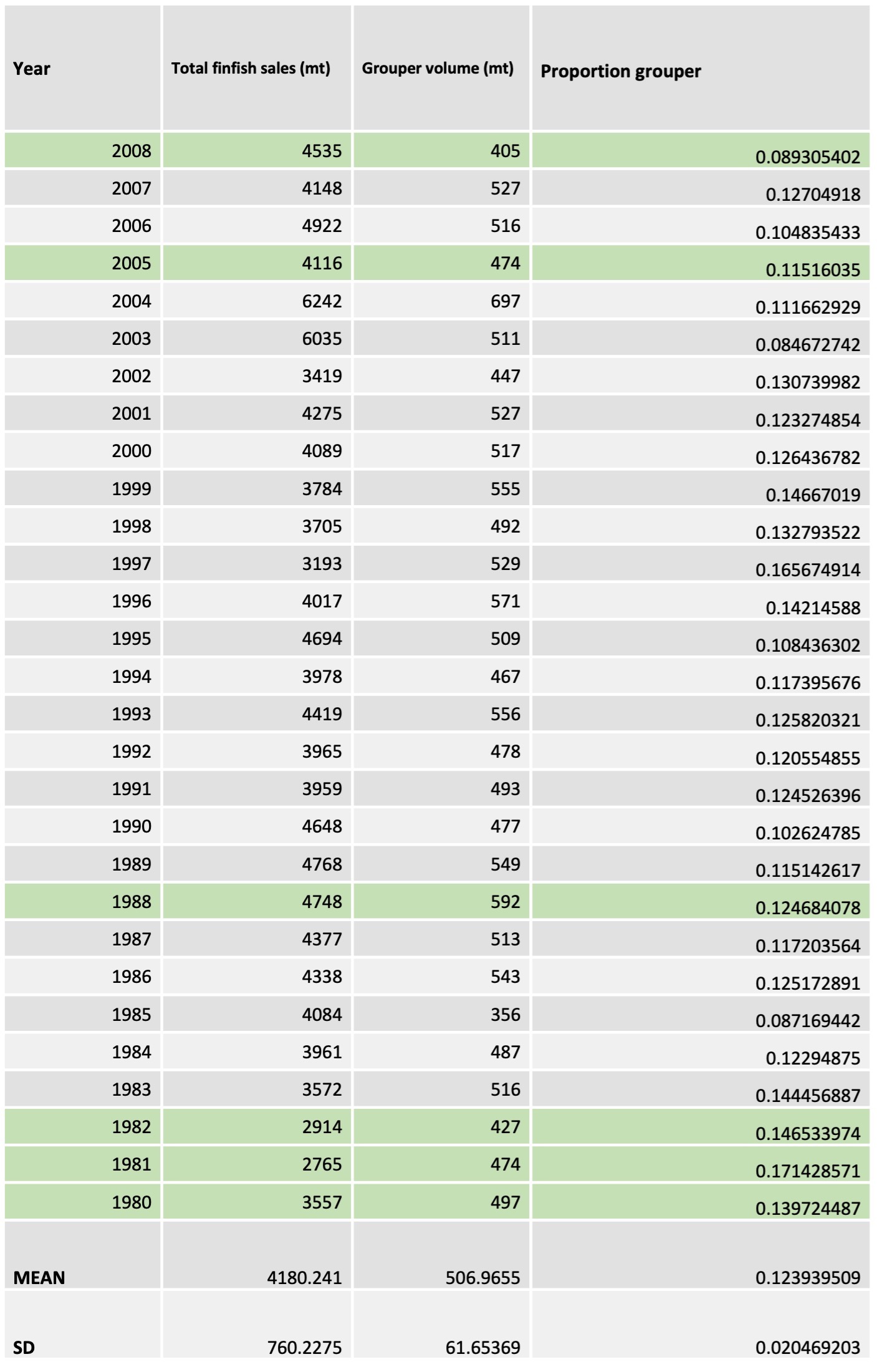

Supplement: Supplementary file 3 — Table S1. Recorded commercial finfish landings (mt) sold across local markets in Fiji, and landings and proportion of landings comprised of groupers, according to government data from 1980 to 2008 (Ministry of Fisheries Annual Reports 2014–2021). The data collection programme ceased after 2008. [file JFB-107-34-s003.jpg]
